# A Systematic Review of Hybrid Polymeric Woven Composites: Mechanical Performance, Numerical Simulation, and Future Perspectives

**DOI:** 10.3390/ma19091887

**Published:** 2026-05-03

**Authors:** Chala Amsalu Tefera, Sławomir Duda, Sebastian Sławski

**Affiliations:** Department of Theoretical and Applied Mechanics, Faculty of Mechanical Engineering, Silesian University of Technology, Konarskiego 18A, 44-100 Gliwice, Poland

**Keywords:** hybrid polymeric woven composites, multiscale, barely visible impact damage, strain rate sensitivity, damage tolerance, impact resistance, constitutive modelling

## Abstract

Hybrid polymeric woven composites (HPWCs) are increasingly important in automotive, aerospace, and renewable energy structures where low weight, impact tolerance, damage containment, and superior mechanical properties are required. By combining dissimilar fibres within woven architectures, HPWCs can achieve a more favourable balance of stiffness, strength, and energy absorption than single-fibre woven systems; however, experimental evidence and predictive modelling remain insufficiently integrated, particularly under dynamic and post-impact loading. This systematically searched critical review provides an HPWC-focused synthesis that links architecture-driven mechanical behaviour, damage development, and multiscale numerical simulation within a single framework. The effects of reinforcement architecture, fibre pairing, and matrix selection on tensile, flexural, compressive, interlaminar, strain rate-dependent, and impact responses are examined, with particular emphasis on barely visible impact damage and post-impact residual strength. Macroscale, mesoscale, and microscale finite element strategies are critically compared in terms of predictive fidelity, computational cost, and suitability for design-orientated assessment. The main contribution of this review lies in integrating experimental characterisation with modelling limitations, validation requirements, and industrial relevance, thereby clarifying where current approaches are effective and where critical gaps remain. Practical implications for lightweight structural design, impact-resistant components, and future validation-driven research are highlighted.

## 1. Introduction

Hybrid polymeric woven composites (HPWCs) are a growing class of high-performance structural materials for applications that require low weight, high specific strength, and enhanced damage tolerance [1,2,3]. In HPWCs, hybridisation generally refers to the intentional use of two or more reinforcement types within a polymer matrix. This hybridisation can be implemented at different length scales (e.g., within yarns, within plies, or across laminate stacks) and may include, but is not limited to, intralayer and interlayer configurations [1,2,3,4]. Because HPWCs are often used under combined mechanical and environmental loading, their long-term damage evolution and durability can be strongly affected by architecture-dependent failure mechanisms, making a mechanistic understanding essential for reliable design.

Woven composites, one of the most widely used types of fibre-reinforced polymers (FRPs), have a higher multidirectional stiffness, impact resistance, and delamination strength than FRPs reinforced with unidirectional or randomly orientated fibres. This is due to their interlaced yarn structures, which provide improved load distribution and design flexibility [5,6,7]. Such advantages have resulted in major use in industry. Structural parts such as wings, fuselages and frames have been made of woven composites in the aerospace industry [1,2]. In the automotive industry, they are used on bumpers, body panels, and interior reinforcements to improve fuel efficiency and crash resistance [1,6]. Similarly, in the case of renewable energy systems, large and complex shaped components, such as wind turbine blades, are manufactured with woven fabric composites because of their ability to conform to complex mould geometries, as well as their high impact resistance [8,9,10]. Such advantages have resulted in major use in industry, with established and emerging applications spanning the aerospace, automotive, wind energy, marine, and construction sectors.

**Figure 1:**
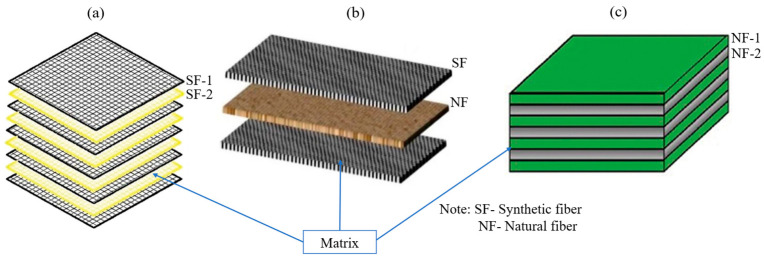
Schematic representation of HPWC architectures: (**a**) hybrid synthetic/synthetic composite combining synthetic fibre 1 and synthetic fibre 2. Reproduced with permission from [15]. (**b**) Hybrid synthetic/natural composite combining synthetic and natural fibres. Reproduced with permission from [18]. (**c**) Hybrid natural/natural composite consists of natural fibre 1 and natural fibre 2 in a polymer matrix.

Recent advances in the design of hybrid reinforcements have increased the functionality of woven composites by incorporating stiff, load-bearing fibres along with more compliant and energy-absorbing reinforcements [5,6,9,11,12]. In general, regardless of hybridisation scale (intralayer, interlayer, or yarn-level), fibre-type combinations in HPWCs can be classified as: synthetic/synthetic (Figure 1a), in order to balance stiffness, strength, and cost [13,14,15]; synthetic/natural (Figure 1b), in order to combine mechanical performance with better sustainability [11,16,17]; or natural/natural (Figure 1c), in order to further reduce environmental impact while managing acceptable structural performance [3,8]. Although natural/natural hybrids remain relevant, the present review focuses primarily on HPWCs incorporating at least one high-performance synthetic reinforcement due to their strong relevance in load-bearing and damage-critical structures.

By combining complementary fibre properties, for example, the high tensile stiffness/strength of carbon fibres with the toughness and impact resistance of aramid or selected natural fibres, HPWCs can achieve improved strength-to-weight ratios, enhanced energy absorption, and broadened multifunctionality while retaining cost effectiveness [3,9,11,12,13,19]. However, hybridisation can also introduce design complexities, including fibre–matrix interfacial compatibility, heterogeneous damage initiation and progression, and challenges in achieving predictive numerical modelling accuracy, particularly under dynamic loading and impact conditions. Establishing robust links between hybrid architecture, damage mechanisms, and simulation fidelity is therefore a key requirement for reliable HPWC design.

In recent years, several review articles have strengthened the foundations for HPWCs and for woven/durable composite reinforcements. Recent reviews have summarised hybrid FRP concepts, processing routes, and performance trade-offs with a specific focus on natural/synthetic hybridisation and sustainability-based design [10,20,21,22]. In parallel, state-of-the-art reviews on woven and 3D woven composites have consolidated the knowledge on textile reinforcement architectures and impact-related damage tolerance, including experimental characterisation and damage assessment practices [10,23]. However, these works generally do not provide a systematic, HPWC-focused synthesis that simultaneously links (i) hybrid woven architecture material selection, (ii) failure and damage mechanisms under quasi-static and strain rate-dependent/impact loading, (iii) BVID and post-impact residual performance, and (iv) validated FEM strategies and limitations within a single, coherent framework. Therefore, a systematically structured review focused on HPWCs that matches experimentally observed damage/residual strength with modelling fidelity and application constraints remains timely.

Consequently, this paper presents a systematically searched critical review informed by a structured literature search of Scopus-indexed publications on HPWCs and answers the following research questions summarised in Table 1. The objectives are to consolidate the existing understanding, to identify limitations and gaps (experimental and numerical), and to propose the direction of future innovation and adoption by industry.

The aim of this review is to consolidate the current understanding of the mechanical behaviour of HPWCs and their predictive numerical modelling. The specific objectives are to: (i) synthesise reported findings on the influence of reinforcement architecture and fibre hybridisation on quasi-static and dynamic mechanical properties; (ii) critically examine strain rate sensitivity, impact response, and post-impact residual strength associated with BVID; (iii) systematically compare finite element modelling strategies across macroscale, mesoscale, and micro-scale in terms of predictive fidelity and computational cost; and (iv) identify key experimental and modelling gaps in order to define priority research directions that support the reliable design and industrial adoption of HPWCs. To make the scope boundaries and organisational logic of the review explicit, Table 2 summarises the framework used to classify the HPWC literature surveyed in this work.

## 2. Review Methodology and Structured Search Process

This article is presented as a systematically searched critical review. A structured database search, predefined eligibility criteria, a Preferred Reporting Items for Systematic Reviews and Meta-Analyses (PRISMA 2020)-informed flow diagram [24], and standardised data extraction were used to support transparent study identification and thematic synthesis across a heterogeneous body of experimental and modelling literature.

### 2.1. Search Strategy and Data Source

The primary literature search was conducted using the Scopus database, selected for its comprehensive coverage of peer-reviewed journals in engineering and materials science, which includes major publishers, e.g., Elsevier, MDPI, Springer, Wiley, Taylor & Francis, and IEEE. To capture research on the mechanical performance and numerical analysis of HPWCs, the search query targeted titles, abstracts, and keywords using the following strategy:

TITLE-ABS-KEY ((“hybrid woven composite*” OR “woven hybrid composite*” OR “hybrid polymeric woven” OR “woven fabric composite*” OR “textile composite*” OR “woven composite*” OR “hybrid fibre reinforced” OR “hybrid fiber reinforced” OR “hybridisation” OR “hybridization”) AND (“polymer” OR “epoxy” OR “thermoplastic” OR “vinyl ester” OR “resin” OR “thermoset”) AND (“mechanical propert*” OR “fatigue behav*” OR “dynamic loading” OR “strain rate” OR “failure mechanism*” OR “barely visible impact damage” OR “BVID” OR “numerical simulation” OR “finite element” OR “damage tolerance” OR “impact resistance” OR “interlaminar” OR “delamination” OR “residual strength”)).

The query was structured using three Boolean AND blocks targeting composite architecture, polymer matrix systems, and mechanical/damage phenomena, respectively. The transformation operators (*) were applied to capture spelling variants and morphological forms.

### 2.2. Inclusion and Exclusion Criteria

**Figure 2:**
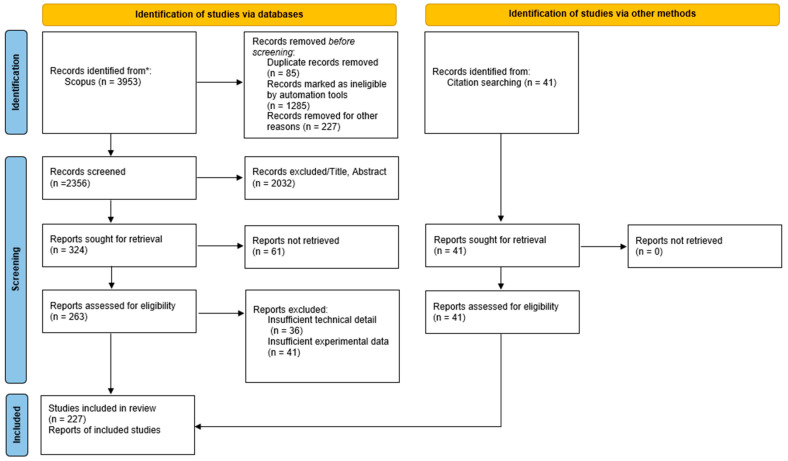
PRISMA 2020 flow diagram for systematic searched critical review literature review. *: Table S1.

The initial search returned 3953 documents. To refine this to a relevant and manageable corpus for in-depth review, the following inclusion criteria were applied: (i) publication year: from 2015 to 2025; (ii) language: English; (iii) subject areas: Engineering, materials science and physics and astronomy; and (iv) document type: Journal articles and review papers. To further refine the dataset, the following exclusion criteria were applied: (i) Conference papers, book chapters, editorials, and patents; (ii) studies not primarily focused on mechanical properties, damage behaviour, or numerical simulation; and (iii) duplicate publications. In addition, the references of every document were manually checked to find relevant documents in the research area. After applying the eligibility criteria, a final corpus of 227 articles was retained for in-depth qualitative synthesis and narrative discussion (Section 4, Section 5 and Section 6) (Figure 2). For bibliometric analysis (Section 3), metadata from Scopus records filtered to the 2015–2025 time window (*n* = 2668) was analysed to map publication trends and research contributions (Table A1, Table A2 and Table A3) [25]; this two-tier strategy reduces selection bias in trend mapping while allowing a deeper critical synthesis of the selected corpus. The PRISMA flow diagram (Figure 2) summarises the record selection steps and associated counts at each stage.

### 2.3. Data Extraction and Analysis

A standardised form was used to extract data from the 227 core publications. The information obtained included: (i) composite material system (matrix, reinforcement, and weave architecture); (ii) key research focus (e.g., reinforcement architecture, impact, fatigue, and hybridisation); (iii) experimental and numerical methodologies; (iv) principal findings related to mechanical performance and damage behaviour; and (v) identified limitations or future work. This structured extraction enabled thematic analysis and identification of convergent trends and persistent gaps in the literature.

## 3. Results of the Bibliographic Analysis

### 3.1. Quantitative Publication Trends

**Figure 3:**
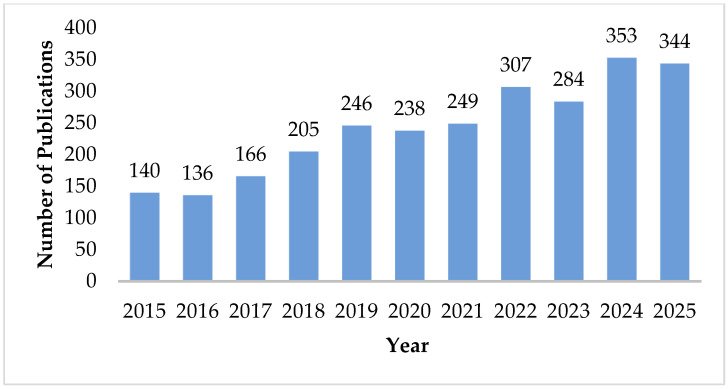
Annual trend in the publication of hybrid and polymeric woven composites (2015–2025).

Research on HPWCs has expanded substantially in the last decade. Based on the Scopus dataset analysed in this review (*n* = 2668 records; 2015–2025), the annual publication output increased from 140 articles in 2015 to 284 articles in 2023, reaching 344 articles in 2025 (Figure 3). The annual publication counts for the entire period are provided in Table A1 [25].

**Figure 4:**
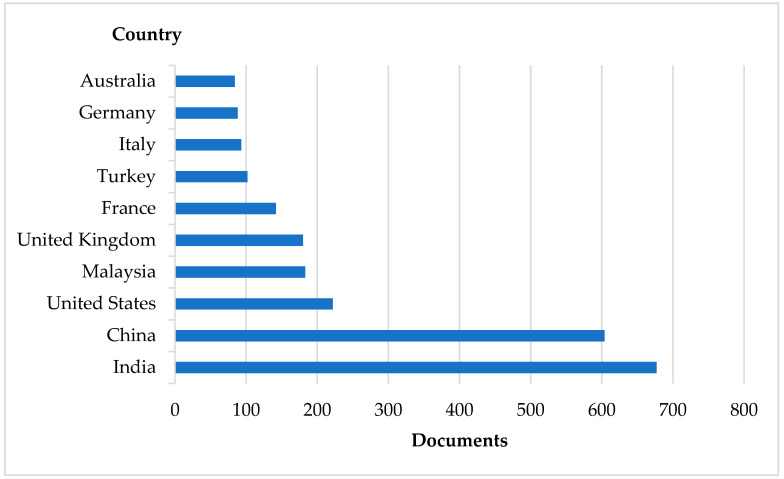
Trend in HPWC publications by country (2015–2025) [25].

The country-level analysis based on author affiliations (Figure 4) indicates a concentrated distribution of the output across a small number of regions. In the dataset presented here (2015–2025; *n* = 2668), India (677 documents; 25.4%), China (603; 22.6%), and the United States (222; 8.3%) were the leading contributors, together accounting for 56.3% of documents. The full country counts and shares are reported in Table A2. Because multi-country collaborations can assign one document to multiple countries, country shares do not necessarily sum to 100%.

At the institutional level, the most frequent contributing organisations include the Ministry of Education of the People’s Republic of China (117 documents; 4.4%), Universiti Putra Malaysia (67; 2.5%), Donghua University (66; 2.5%), and CNRS (Centre National de la Recherche Scientifique) (50; 1.9%) (Table A3) [25]. The leading publication venues were Composite Structures (143 documents; 5.4%), Polymer Composites (130; 4.9%), and Materials Today: Proceedings (93; 3.5%) (Table A4), confirming that HPWC research is primarily disseminated through core composites and materials engineering outlets.

### 3.2. Thematic Evolution and Research Focus

#### 3.2.1. Research Clusters and Emerging Topics

**Figure 5:**
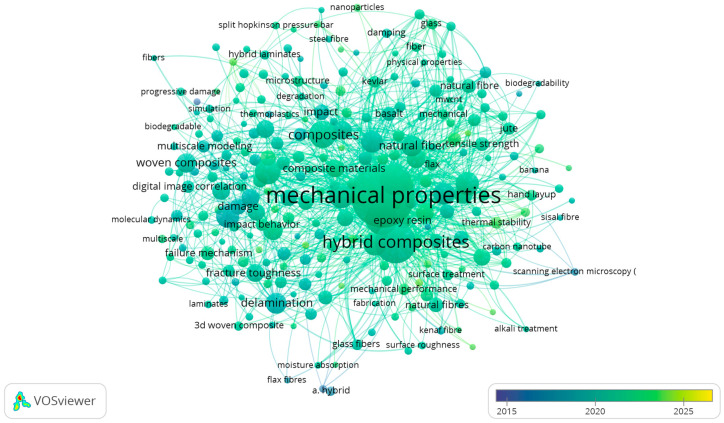
The co-occurrence network of keywords has been visualised using VOSviewer, showing the main research groups in HPWCs.

To map the intellectual structure of HPWC research, a keyword co-occurrence network was constructed using VOSviewer 1.6.20 (Figure 5) [26]. Analysis was performed using author keywords (with index keywords used only when author keywords were unavailable), and synonym harmonisation was applied using a thesaurus file (e.g., merging the “finite element method” and “finite element analysis” and standardising “BVID” as “barely visible impact damage” where applicable). Keywords were included if they occurred at least five times in the dataset, resulting in 130 terms in the final network. Co-occurrence links were computed using full counting, and normalisation was performed using the association-strength method (VOSviewer default). The overlay visualisation used the average publication year to indicate thematic evolution (blue = earlier; yellow = more recent):

- Core mechanical performance (established cluster): A central, blue-coded cluster groups keywords such as “strength,” “impact,” “delamination,” and “fatigue,” representing the enduring foundation of experimental mechanical characterisation.
- Computational advancements (emerging cluster): Keywords such as “finite element analysis,” “multiscale simulation,” and “damage analysis” appear in recent (yellow/green) hues, visualising the field’s shift toward predictive modelling to complement experimental work.
- Sustainable materials (growing trend): Terms like “natural fibre,” “jute,” and “hybrid woven composites” highlight contemporary trends. Their integration with core mechanical keywords shows an active assessment of the performance of sustainable materials.

#### 3.2.2. Evolution of the Focus of the Research

**Figure 6:**
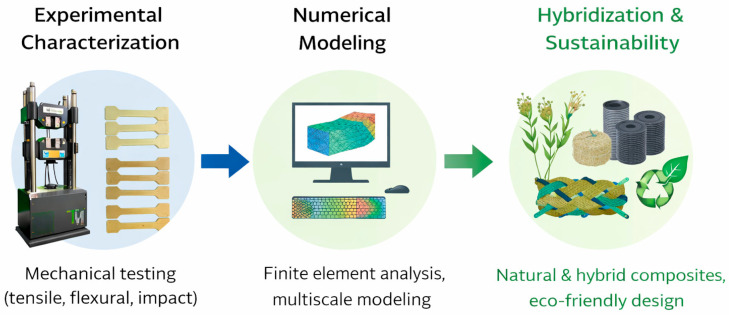
Conceptual evolution of the research focuses from experimental characterisation to computational modelling and sustainable hybrid design.

During the past decade, HPWC research has progressed through three broad themes: foundational experimental characterisation [27,28,29,30,31,32], increasing adoption of numerical simulation [33,34,35,36], and sustainability-orientated hybrid design [37,38,39]. Initial research established baseline mechanical performance by tensile, flexural, and impact tests and provided important reported damage mechanisms in aerospace, automotive, and construction applications [15,27,28,40]. Subsequently, computational approaches, including FEA and multiscale modelling, were increasingly used to interpret experiments, explore parameter sensitivity, and support design studies under complex loading conditions [41,42,43]. More recently sustainability-orientated innovation has gained pace, such as natural and synthetic fibre hybridisation strategies to minimise environmental impact and pursue acceptable mechanical performance [28,44,45,46,47,48,49]. Simultaneously, research is also growing into the fields of bio-based matrices and recyclability-focused pathways (e.g., bio-resins and recycling schemes) to deal with end-of-life issues [37,38,47,50,51,52]. This thematic progression is summarised in Figure 6.

## 4. Mechanical Properties, Damage Mechanisms, and Durability of HPWCs Under Various Influencing Factors

**Figure 7:**
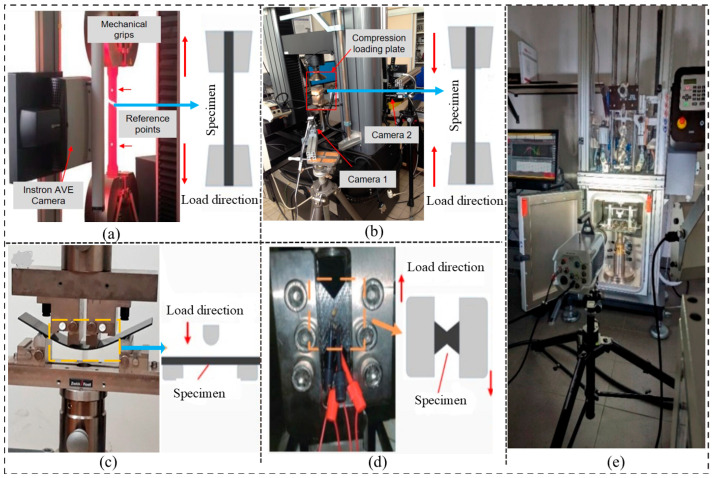
Experimental test methods: (**a**) tensile test and (**b**) compression test. Adapted from ref. [58]; (**c**) flexural test; (**d**) shear test; (**e**) impact damage tolerance using CAI. Adapted from ref. [59].

The mechanical properties of HPWCs are influenced by various factors. Comprehensive experimental studies have been conducted to characterise the mechanical behaviour, damage mechanisms, and durability of HPWCs in quasi-static and dynamic tests. Figure 7 displays some of the ISO-based experimental test methods, including tensile testing (ISO 527-4) [53], flexural testing (ISO 14125) [54], compression testing (ISO 14126) [55], shear testing (ISO 14129) [56], and impact damage tolerance using compression after impact (CAI) procedures (ISO 18352) [57]. These standardised methods support consistent comparison of mechanical performance across architectures and hybridisation strategies and provide reliable benchmarks for numerical model calibration and validation. Taking into account previous research, this section synthesises experimental evidence on the influence of key factors such as reinforcement architecture, hybrid fibre concepts, strain rate effects, and damage progression. The focus is on establishing the deformation and failure mechanisms critical for structural performance and the development of predictive models.

### 4.1. Influence of Reinforcement Architecture

The reinforcement architecture is one of the main factors that determines the mechanical response of HPWCs. Unlike unidirectional laminates, the performance of woven composites is based on the interlaced yarn structure, such as the orientation of the yarn, the crimp, the nesting, and the interactions between all yarns that jointly affect the evolution of stiffness, strength, and damage [60,61]. Studies show that weave architecture directly controls load transfer efficiency, stress redistribution, and dominant failure mechanisms [52,62].

Among conventional two-dimensional (2D) woven architectures, plain weave has the highest frequency of interlacing, which makes it have excellent dimensional stability and resistance to yarn slippage. However, high yarn crimp results in lower tensile and compressive strength compared to other weave types [32,61,62]. Chowdhury et al. [31] underlined that despite reduced strength, plain woven composites excel in impact resistance and interlaminar integrity, making them appropriate where damage tolerance is required rather than maximum in-plane stiffness.

Twill weaves have less crimp angle and greater float length than plain weaves, which improves the alignment of the fibres and the transfer of loads under tensile and flexural loads. Sahbaz K. [63] and Huang J et al. [64] reported that twill-woven composites often have higher tensile strength, flexural modulus, and fatigue resistance, and slightly reduced impact resistance due to lower interlacing density. Satin weaves, characterised by the longest float lengths and least crimp, favour near unidirectional alignment of the fibres and have high tensile and flexural characteristics. Axinte A et al. [65] showed that, in the yarn-dominated directions, satin woven composites may approach the stiffness and strength of unidirectional laminates while retaining multidirectional reinforcement benefits; however, satin architectures do not replicate UD performance across all directions or loading modes. However, longer floats can lead to the appearance of localised stress concentrations and reduced resistance to transverse cracking and delamination under compressive and out-of-plane loading [32,66].

**Figure 8:**
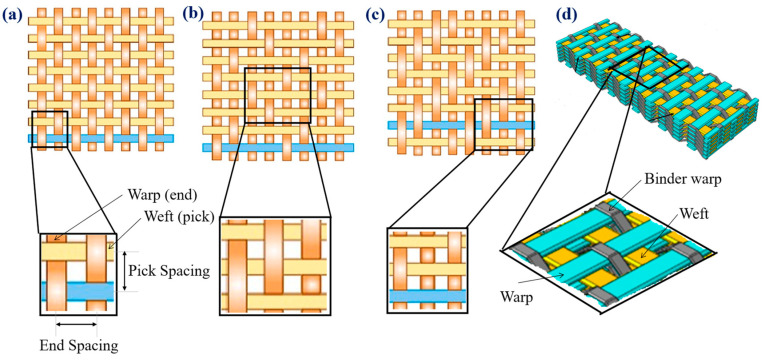
Schematic illustrations of different types of weaves in HPWCs: (**a**) Plain weave with 1:1 yarn interlacing; (**b**) twill weave with diagonal patterns for better flexibility and load transfer; and (**c**) satin weave with long yarn floats for better fibre alignment. Reproduced from ref. [10] (**a**–**d**) 3D woven layer-to-layer angle-interlock fabric. Reproduced from ref. [68].

Beyond traditional 2D fabrics, three-dimensional (3D) woven architectures, such as orthogonal, angle-interlock, and layer-to-layer, provide enormous improvements through thickness performance. Binder yarns in 3D systems improve the strength of interlaminar shear and effectively inhibit the propagation of delamination [67]. These architectures exhibit better damage tolerance under impact, post-impact compression, and fatigue loading than laminated 2D woven composites [63,67]. However, incorporating through-thickness (binder) yarns can reduce the volume fraction of fibre in the plane and introduce waviness (crimp), which can lead to modest reductions in tensile and flexural stiffness compared to 2D laminates [11,63]. Figure 8 schematically shows the main weave architectures that are used in HPWCs, such as plain, twill, satin, and 3D woven configurations with different yarn interlacing patterns and structural characteristics. These architectural disparities are shown directly in differentiated variations in the mechanical response and the progression of damage evolution.

**Figure 9:**
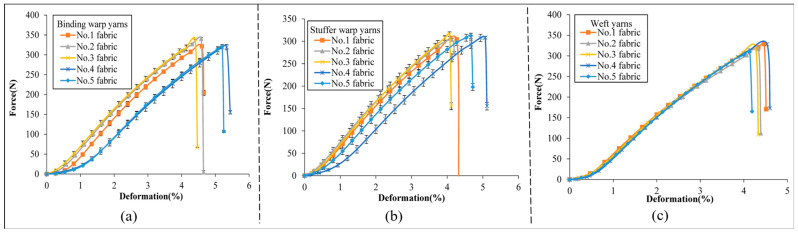
Architecture-dependent tensile force–deformation response of single yarns after weaving in different 3D warp interlock fabrics (3DWIFs): (**a**) binding warp yarns, (**b**) stuffer warp yarns, and (**c**) weft yarns. Reproduced from ref. [68].

Architecture also affects the tensile response of individual yarn systems after weaving, particularly in 3D warp interlock fabrics (3DWIFs). Figure 9 presents representative tensile force–deformation curves for single yarns extracted after weaving from different 3DWIF architectures, including binding warp, stuffer warp, and weft yarns. The Figure shows that the yarn-scale mechanical response is architecture-dependent, with clear variations in the initial deformation stage, peak tensile force, and failure deformation among the different fabric variants. These differences are associated with the distinct yarn paths imposed by the interlock design, especially the degree of yarn waviness and crimp generated during weaving. In the corresponding study, five 3DWIF architectures with different binding warp evolutions but identical warp and weft densities were experimentally compared, and the authors demonstrated that fabric architecture influences tensile behaviour in both the warp and weft directions. Therefore, the results highlight that reinforcement architecture governs not only laminate-scale stiffness and damage development, but also the mechanical efficiency of individual yarns after weaving, which ultimately contributes to the overall tensile and failure response of HPWCs [68].

The reinforcement architecture further controls compressive behaviour, where the crimp of the yarn and the misalignment of the fibre influence the micro-buckling and kink-band formation. High-crimp architectures, such as plain weave, typically display low compressive strength, and satin and optimised twill weaves have demonstrated increased resistance due to improved fibre straightness [6,32,61]. Interlaminar properties such as delamination resistance and interlaminar shear strength are highly affected by weave topology, yarn nesting, and the formation of resin-rich areas during manufacturing [36,61].

### 4.2. Hybrid Reinforcement

Hybrid reinforcement is a material design strategy approach to tailor the properties of woven composites. By combining fibres with complementary characteristics, engineers optimise performance metrics, such as stiffness, toughness balance, impact resistance, cost efficiency, unattainable with single fibre systems [14,42,48,49,69]. This section analyses hybridisation strategies, focusing on fibre selection, architectural implementation, and the resulting mechanical performance.

#### 4.2.1. Synthetic Fibre Hybrid Woven Composites

Glass/carbon fibre systems have been widely investigated, in which high modulus carbon provides stiffness and load-bearing capacity, while glass ensures strain to failure, damage tolerance, and cost savings [70,71]. Experimental investigations consistently demonstrate that glass/carbon hybrid woven composites have exhibited progressive failure and better damage tolerance than monolithic carbon systems, particularly under flexural and impact loads [28,29,70,71,72]. The response is strongly influenced by the placement of the fibre types: carbon-rich outer layers will maximise the bending stiffness and strength, while glass-rich core layers will improve energy absorption ability and delay catastrophic fracture by more gradual damage evolution [58,70,72].

Aramid-based hybrid woven composites, which are usually used with carbon or glass fibres, are used mainly to improve impact resistance, fracture toughness, and post-impact integrity. Due to their high tensile strength and energy dissipation, they effectively suppress crack propagation and fibre breakage under dynamic loads [73,74,75,76]. Carbon/aramid hybrids suppress the brittle failure mechanisms, enhancing residual strength following impact events [73]. However, the compressive ability of aramid is limited by its low compressive strength and tendency to fibrillation [30]. In addition, interfacial incompatibility and moisture sensitivity can reduce long-term durability, which highlights the need for careful material selection and appropriate surface treatments.

Ultra-high-molecular-weight polyethylene (UHMWPE) has attracted growing interest due to its excellent specific strength, toughness, and impact/ballistic resistance. When hybridised with stiffer fibres (carbon or Kevlar), layers of UHMWPE can enter energy absorption and damage diffusion under high-strain-rate loading to enhance impact tolerance without sacrificing areal density [77,78,79]. However, weak interfacial bonding, high viscoelasticity, and temperature sensitivity can cause less efficient load transfer and even make predictive modelling more difficult, particularly under dynamic conditions [78,80,81]. Consequently, the hybrid design usually demands interface designing (e.g., surface modification, coupling strategies), as well as thoughtful choices of stackings to balance stiffness and impact performance.

Basalt fibres offer an interesting hybrid potential due to intermediate stiffness, good thermal stability, and lower cost than carbon fibres [82]. Carbon/basalt and glass/basalt composites have shown better resistance to impact and flexural performance than pure glass systems, while maintaining better cost efficiency than carbon-dominated laminates [29,83]. Providing a balance of performance and affordability for infrastructure, transportation, and energy-related applications.

#### 4.2.2. Natural/Synthetic Hybrid Woven Composites

Driven by sustainability goals, natural/synthetic hybrid woven composites integrate plant-based fibres (e.g., flax, hemp, jute, and kenaf), which reduce the synthetic fibre fraction while maintaining relevant mechanical performance for application [48,50,84]. In practice, the hybrid effect is strongly configuration-dependent, because stacking sequence and the hybridisation mode (interply vs. intraply/co-woven) can produce either positive or negative outcomes depending on where the stronger plies are placed and how loads are transferred across interfaces [46,50].

**Figure 10:**
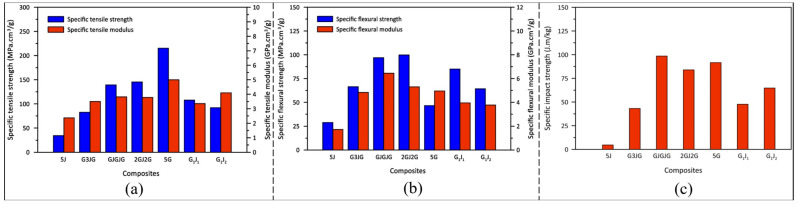
Specific mechanical properties of pure glass/epoxy, pure jute/epoxy, and glass/jute hybrid composites: (**a**) specific tensile strength and modulus; (**b**) specific flexural strength and modulus; and (**c**) specific impact strength. Reproduced from ref. [46].

A controlled quantitative example is provided by the woven jute/glass/epoxy laminates reported by Altaee and Mostafa [46], from which the specific tensile, flexural, and impact properties in Figure 10 have been adapted. In that study, the authors compared pure jute (5J), pure glass (5G), interply hybrids (G3JG, GJGJG, and 2GJ2G), and intraply hybrids (G1J1, G1J2), where G denotes a glass-fibre ply, J denotes a jute-fibre ply, and numerical prefixes indicate the number of consecutive plies of that fibre type (e.g., 2GJ2G = two glass plies/one jute ply/two glass plies). They showed that increasing the glass fraction in the interply systems improved tensile performance, while the two intraply architectures exhibited similar tensile moduli but different tensile strengths; notably, the tensile strength of G1J1 was reported to be ~41% higher than that of G1J2, highlighting that yarn-level hybridisation strategy (not only fibre type) can significantly influence mechanical outcomes. In flexure, the author further reported that 2GJ2G provided the highest flexural properties in their dataset, followed by GJGJG, which they related to classical bending mechanics, where the outer layers experience the highest axial stresses. These results support a general review conclusion: flexural efficiency can be enhanced when higher-stiffness plies are strategically placed away from the neutral axis, while natural/fibre plies in the core can contribute to different damage-development pathways rather than immediate catastrophic fracture [46,85].

However, impact-related interpretations must be stated carefully. The authors demonstrated that the Charpy impact strength (energy absorbed per unit cross-sectional area, kJ/m^2^) of 2GJ2G and GJGJG were lower than that of pure glass by ~4% and ~16%, respectively, showing that “glass layers always improve impact resistance” is not universally valid [46]. A more accurate interpretation is that glass plies can, in some configurations, change the stress distribution and constrain deformation in the natural fibre region and, in turn, influence the way in which damage develops. For example, Khalid et al. [86] reported microscopy-based evidence that an outer glass layer can help minimise stress redistribution in jute layers in glass/jute hybrids. Beyond mechanical testing, durability studies reinforce a similar “protective-skin” concept under environmental exposure: Calabrese et al. [87] found that outer glass layers in flax/glass hybrid epoxy laminates acted as a protective shield during humid/dry cycling, stabilising mechanical performance and supporting recovery during the dry phase.

Evidence from other woven hybrid systems confirms that stacking architecture governs damage tolerance and residual performance. For example, hybrid laminates based on twill-woven flax/basalt fabrics revealed that the impact resistance and residual strength of CAI strongly depend on the stacking configuration, with different damage mechanisms observed with various layouts. Similarly, interlayer woven flax/basalt/glass hybrids indicate that the tensile/flexural/impact behaviour is controlled by the combination of the reinforcements and the ply stacking sequence, which highlights the need to consider hybrid-woven design as architecture-specific systems rather than assuming the uniform “glass-skin” benefits over all the loading scenarios [23,85].

Despite these advantages, the performance of natural/synthetic hybrid woven composites is often limited due to fibre–matrix interfacial adhesion and moisture sensitivity, which can lead to scattering of the properties and to lower long-term durability [50,87,88]. Consequently, surface treatments, matrix modification, and architecture optimisation are constantly found to be crucial steps to achieve reliable performance in semi-structural and load-bearing applications [46,48,84,86,87].

#### 4.2.3. Comparative Performance and Design Considerations

From a performance standpoint, the combination of fibre strength with the enhanced dynamics of hybrid reinforcement allows us to realise synergistic mechanical behaviour via hybrid reinforcement that cannot be achieved with single-fibre systems. The comparative behaviour of hybrid systems should be interpreted in light of the differing stiffness, strength, strain-to-failure, and density ranges of the constituent fibres, which govern their load-sharing roles and failure sequences within the woven architecture. Glass/carbon hybrids generally have better tensile strength and flexural stiffness than pure glass fibre composites, with great savings in cost over carbon-based systems [28,29,49]. Aramid/carbon hybrids exhibit superior impact resistance and post-impact integrity characteristics and may have lower compressive loading capacity than carbon-dominated laminates [73]. The natural/synthetic hybrids offer tangible environmental and economic advantages in that their long-term durability is not as good as that of fully synthetic composites. Hybrids based on UHMWPE have excellent lightweight and impact resistance, but are sensitive to temperature and humidity and interfacial deterioration, leading to loss of mechanical reliability in demanding service environments [17,46,89].

In general, while hybrid reinforcement concepts have significant benefits in terms of performance modification and multifunctionality, their successful implementation needs careful consideration of fibre compatibility, interfacial behaviour, manufacturing complexity, and loading conditions depending on the application. These challenges highlight the importance of integrated experimental and numerical investigations that can capture hybrid-specific damage mechanisms and fill the gap towards optimal hybrid composite design. Table 3 shows an overview of hybrid reinforcement systems in HPWCs and their dominant mechanical characteristics.

From a design standpoint, fibre pairing and spatial arrangement should be selected according to the dominant loading scenario and the desired failure mode. Carbon/glass hybrids are particularly attractive when a balance between stiffness, impact tolerance, and cost efficiency is required, whereas carbon/aramid systems are more favourable when energy absorption, crack arrest, and post-impact integrity are priority objectives [58,73,89]. Likewise, 3D woven architectures are preferable when delamination suppression and through-thickness load transfer govern performance, while 2D twill or satin systems remain advantageous where in-plane efficiency, manufacturability, and lower modelling complexity are more important [23,62,90,91]. These relationships indicate that HPWC design should not be based on constituent properties alone, but on the combined effects of architecture, hybridisation strategy, and the targeted damage-tolerance requirement [29,66,85].

**Table 3:** Overview of hybrid reinforcement systems in HPWCs.

Hybrid Fibre System	Primary Mechanical Advantage	Main Limitation	Dominant Loading/Performance Benefit	Typical Application Domain	Ref.
Glass/Carbon	Balanced stiffness-cost performance; improved damage tolerance compared to CFRP	Interfacial stress mismatch; reduced design simplicity	Flexural stiffness, impact resistance	Automotive structures, wind energy	[28,58,70,71]
Glass/Carbon/Aramid (Kevlar)	Synergistic stiffness, strength, and impact resistance; personalised deformation response	Increased manufacturing complexity; interface management	Stress redistribution, deformation control, impact tolerance	Aerospace panels, advanced structural components	[89]
Carbon/Basalt/Glass	Improved flexural performance with improved cost efficiency	Lower stiffness than fully carbon systems	Flexural stiffness to cost optimisation	Automotive and civil structures	[29,83]
Carbon/Aramid	Enhanced impact resistance and fracture toughness; reduced brittle failure	Limited compressive strength; moisture sensitivity	Post-impact residual strength, energy dissipation	Aerospace panels, protective structures	[73]
Carbon/UHMWPE	Exceptional specific strength and energy absorption	Weak fibre–matrix bonding; viscoelastic effects	High-strain-rate and ballistic performance	Defence, impact-resistant structures	[33]
Glass/Basalt	Improved thermal stability and durability; moderate cost	Lower stiffness than carbon-based hybrids	Fatigue resistance, thermal loading	Marine and infrastructure	[29]
Glass/Flax	Reduced density and environmental impact; improved damping	Moisture sensitivity; property variability	Vibration damping, low to moderate loads	Automotive interiors, panels	[44]
Carbon/Flax	Improved sustainability with retained stiffness	Interface degradation; reduced durability	Lightweight semi-structural components	Automotive, sports equipment	[92]
Flax/Jute	Low cost, renewable, and biodegradable	Low strength; high moisture uptake	Non-load-bearing and low-impact applications	Building materials and consumer products	[51]

### 4.3. Strain Rate and Dynamic Loading Response

#### 4.3.1. Strain Rate Sensitivity, Viscoelasticity, and High-Speed Testing

The sensitivity to strain rate, viscoelasticity, and high-speed deformation are important factors affecting the mechanical performance of HPWCs. Unlike quasi-static loading, in which deformation and damage occur slowly, with dynamic loading, the materials subjected to the loading are exposed to a rapid strain rate, inducing a time-dependent response of the polymer matrix, which will modify the stress transfer between yarns [93,94]. These effects are especially relevant in structural applications within the aerospace, automotive, and defence industries, where components are regularly subjected to impact, crash, and high-speed deformation scenarios [9,19,76,95].

The sensitivity of the strain rate is the variation in the mechanical properties with the rate of deformation, which depends on the viscoelastic nature of the polymer matrices. As the strain rate increases, the stiffness and strength of polymeric woven composites generally increase due to the reduction in molecular mobility. However, this is often accompanied by a reduction in ductility and dissipation of plastic energy to produce more localised damage and sudden failure [94,96]. In woven architectures, factors such as yarn crimp, interlacing density, and frictional interactions play a role in load redistribution during rapid deformation [89].

In HPWCs, strain rate effects are more complex due to the coexistence of different elastic moduli, different failure strains, and different rate-dependent behaviours of fibres. Hybrid systems that combine stiff, brittle fibres (e.g., carbon) with more ductile reinforcements (e.g., glass, aramid, or UHMWPE) help to mitigate the strain rate-induced brittleness. This is done by encouraging progressive damage mechanisms and delaying catastrophic fracture. The efficiency of this synergy is dependent on the hybrid configuration, the fibre volume fraction, and the arrangement in space, which influence the propagation of the stress waves and the load transfer at the hybrid interface at a high strain rate [93,97].

Viscoelasticity plays a central role in the dissipation of energy under dynamic loading. At higher strain rates, the decrease in the ability of the matrix to experience viscous flow limits crack blunting, which promotes matrix cracking and interfacial debonding. Meanwhile, the interaction between the fibre and the matrix changes with increased interfacial stresses that could accelerate the onset of damage [98]. In hybrid woven composites, variations in the chemistry and stiffness of the fibre surface can be used to enhance or degrade dynamic performance, depending on the quality of the interfacial bonding and the compatibility of the fibre [99]. Under high-speed deformation, like with impact events, strain rate sensitivity has a major influence on the failure mechanism and energy absorption. Higher strain rates may increase energy absorption by fibre stretching, pulling, and frictional sliding, especially with hybrids that involve fibres with high elongations. However, poorly optimised hybrid systems can experience premature failure at fibre interfaces or stress concentration zones, limiting their dynamic performance [76,93,94].

Overall, the strain rate-dependent behaviour of HPWCs is manifested through the synergy of the matrix viscoelasticity, hybridisation of fibres, and woven architecture. Detailed knowledge of these interactions is important for the design of composites with stable dynamic performance.

#### 4.3.2. Resistance to Dynamic Loads

The dynamic load resistance of HPWCs is determined by their response to impact loading, which can be under low- or high-velocity conditions depending on the application. These materials are frequently encountered in transient forces in collision, crash, or ballistic situations, where energy absorption while maintaining structural integrity is of importance [94,97]. Low-velocity impact (LVI) events are typically associated with localised bending, matrix cracking, yarn debonding, and delamination at moderate deformation rates [30,96,100]. In comparison, high-speed impact (HVI) causes rapid propagation of stress waves, fibre fracture, and possible penetration or perforation, so special energy dissipation mechanisms should be used [97,101]. In practice, LVI in structural composite testing commonly spans impact energies in the ~10–50 J range (e.g., tool-drop or service impacts), whereas HVI/ballistic assessments are typically reported using ballistic-limit metrics such as V50 and specific energy absorption, which depend on projectile type and test protocol [102,103].

**Figure 11:**
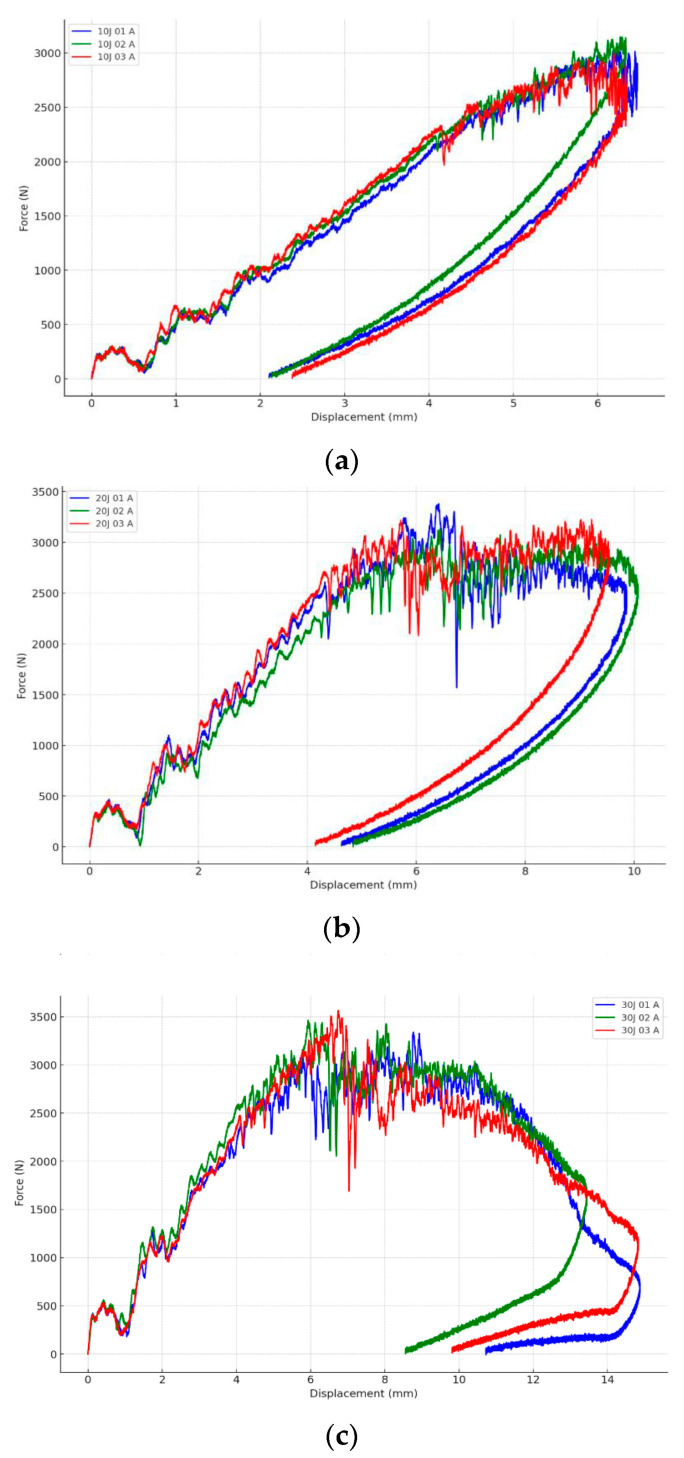
Representative low-velocity impact (LVI) force–displacement responses of hybrid woven composite laminates at (**a**) 10 J, (**b**) 20J, and (**c**) 30 J. Reproduced from ref. [102].

**Figure 12:**
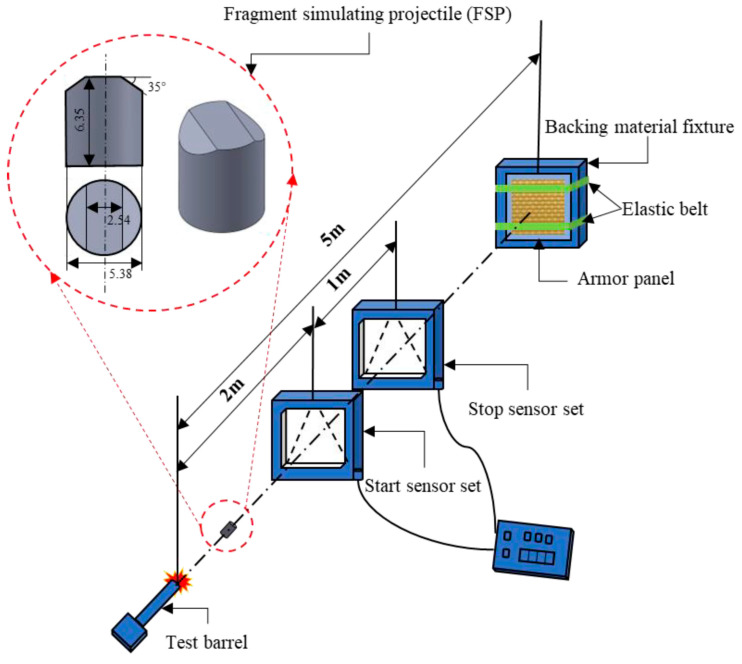
Schematic diagram of ballistic limit V50 test methodology. Reproduced from ref. [103].

To provide quantitative benchmarks for LVI, Figure 11 presents a representative response to impact force–displacement at 10, 20, and 30 J for hybrid woven laminates, illustrating how peak force and deformation evolve with increasing impact energy [102]. For high-velocity/ballistic loading, Figure 12 summarises the standard V50 ballistic-limit test methodology commonly used to define the velocity at which the probability of perforation is 0.5, enabling consistent comparison of ballistic performance across woven composite systems [103].

Under LVI conditions, energy absorption is controlled primarily by matrix-dominated damage processes, such as microcracking, interlaminar delamination, and frictional sliding between interlaced yarns [32,96]. Woven architectures with higher interlacing density, such as plain weaves, generally show improved resistance to delamination due to better interlock of the yarn and constrained crack growth [62,98]. In contrast, twill and satin weaves generally have a better impact performance, i.e., higher energy absorption and lower peak force through the lower crimp thickness of the yarn and more effective stress redistribution during the dynamic bending process [99]. In hybrid woven systems, stiffness mismatches between fibre types can affect delamination patterns and damage propagation during LVI events [30,96,100].

In HVI conditions, matrix deformation is less important, and energy dissipation is dominated by stretching, rupture, pull-out, and frictional interactions of fibres. Hybrid reinforcement strategies are especially helpful in this situation, as the combination of high-stiffness fibres such as carbon or glass and high elongation fibres such as aramid or UHMWPE materials stimulates progressive failure, enhancing energy absorption [104,105]. The local arrangement of the fibres is of prime importance. Ductile fibres with an impact-facing side or in outer layers of fabrics will improve ballistic resistance and decrease back-face deformation [106,107].

The polymer matrix system has an effect on the resistance of dynamic loads by controlling the interlaminar shear strength, crack initiation, and damage evolution. Thermoset matrices have high stiffness and low energy dissipation capabilities, and thermoplastic matrices have high toughness and rate-dependent strain, enhancing impact resistance and damage tolerance [108,109]. In the case of hybrid woven composites, the matrix must be able to accommodate differences in the surface chemistry and deformation behaviour of the fibres to ensure adequate load transfer under dynamic conditions [108,110,111].

Standardised experimental methods are important for dynamic performance assessment and for comparative evaluation of material systems. LVI behaviour is usually measured with drop-weight impact tests to the standards in the ASTM and ISO series, and gives information such as absorbed energy, peak contact force, and damage area. High velocity and ballistic capability are described by gas gun- or projectile-based testing, which focuses on penetration threshold, residual velocity, and specific energy absorption. Although these approaches give useful benchmarks, different details in specimen geometry, boundary conditions, and the configuration of the impactor often do not allow direct comparisons, and there is a need for standardised protocols for testing [107,112].

From a quantitative viewpoint, absorbed impact energy is commonly estimated from the area under the force–displacement response, $E_{abs}=\int \;F\;d\delta$, while ballistic resistance is frequently summarised by the V50 parameter, which defines the impact velocity corresponding to a 50% probability of perforation [102,103]. In general, increasing impact velocity shifts the dominant damage mode from matrix-dominated cracking, interfacial debonding, and delamination toward fibre-dominated rupture, pull-out, frictional sliding, and penetration. Likewise, increasing the fibre volume fraction or placing higher-stiffness fibres in the outer layers typically raises initial stiffness and peak impact force, although the resulting damage morphology remains strongly dependent on weave topology, hybrid layout, and interface quality [97,103]. For this reason, these quantities should not be interpreted as isolated material constants, but rather as architecture- and configuration-sensitive descriptors of dynamic structural response.

Comparative studies have shown positive results in the damage tolerance and energy absorption of HPWCs compared to non-hybrid systems, especially when dynamic loading is applied. Glass/carbon hybrid materials generally have better impact resistance and less brittleness than monolithic carbon materials [70,113], and aramid/carbon materials tend to have better post-impact integrity, but often at the expense of compressive strength [103]. Natural fibre-based hybrids, such as flax or jute, have lower absolute impact resistance but offer sustainability and cost benefits for semi-structural properties [114].

Overall, the dynamic resistance of HPWCs is governed by the coupled interaction of impact velocity, woven architecture, hybridisation strategy, and matrix/interface behaviour. At lower impact velocities, response is dominated by localised bending, matrix cracking, interfacial debonding, and delamination, whereas at higher velocities, energy dissipation increasingly depends on fibre stretching, rupture, pull-out, frictional sliding, and penetration resistance. Architecture determines how this damage localises or redistributes: tightly interlaced or through-thickness reinforced systems can suppress delamination growth, while lower-crimp 2D architectures may improve in-plane efficiency but remain more sensitive to architecture-specific damage evolution. Hybridisation modifies this sequence further by combining stiff load-bearing fibres with more compliant or energy-absorbing reinforcements, which can promote progressive failure and improve post-impact integrity when the fibre arrangement is properly controlled. These interactions explain why impact tolerance in HPWCs cannot be inferred from constituent properties alone but must instead be interpreted as an architecture- and configuration-dependent structural response that is directly relevant to both material selection and predictive modelling.

### 4.4. Damage and Residual Strength

Evaluating damage evolution and post-impact residual strength is of great importance for the determination of the long-term reliability and structural safety of polymeric woven composites in impact-prone environments. In high-performance industries such as aerospace, automotive, and defence, composite structures often endure accidental impacts that may not result in obvious visual damage but may cause serious damage to the mechanical performance. Post-impact behaviour, in particular the occurrence of BVID, the underlying damage mechanisms, and the associated residual strength degradation, is becoming a critical design and maintenance issue, as shown by numerous experimental and analytical studies [115,116].

#### 4.4.1. Barely Visible Impact Damage and Post-Impact Strength

BVID is subsurface damage that is not easily detected by visual inspection but seriously affects the structural integrity and load-carrying capacity of composite materials. In HPWCs, BVID is usually caused by LVI, leading to cracking of the matrix, debonding of the fibre–matrix, and delamination without obvious surface indentations [100,117]. Although the symptom of BVID is very subtle, BVID can act as a precursor of progressive damage accumulation and premature failure under subsequent static, cyclic, or compressive loading [118,119,120,121].

The influence of BVID on residual strength is especially strong in compression-dominated loading cases, where impact-induced fibre misalignment and delamination significantly diminish compression after impact (CAI) strength [100,115,122]. While monolithic woven composites tend to show a sudden post-impact strength degradation, several studies have shown that the post-impact integrity can be improved by the use of hybrid reinforcement, which allows for the progressive evolution of damage [123]. For instance, while Katunin A et al. [115] reported brittle post-impact failure in carbon-dominated systems, the study by Rezasefat M et al. [30] underlines that the addition of fibres such as aramid or natural fibres improves the residual strength by crack-bridging and energy-dissipating mechanisms. Nevertheless, the effectiveness of hybridisation depends highly on the fibre arrangement, interfacial compatibility, and stress redistribution within the weave architecture [37,47,89,124]. Table 4 shows the general overview of BVID effects on the residual strength of HPWCs.

#### 4.4.2. Damage Mechanisms and Fracture Morphology

**Figure 13:**
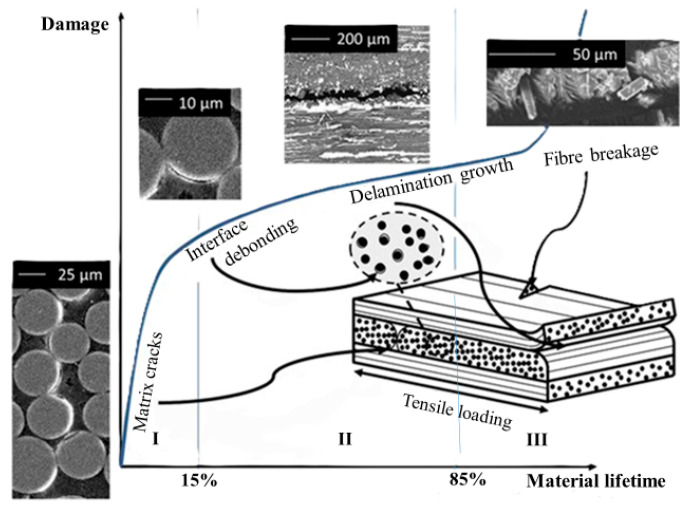
Conceptual schematic of the progressive evolution of damage in polymer composite laminates showing a qualitative change from (**I**) cracking/debonding of the matrix to (**II**) growth of the delamination and interaction of damage and (**III**) fibre breakage (fracture/pull-out) (Concept synthesised from Refs. [36,130,131,132]).

Damage mechanisms in HPWCs are intrinsically multi-modal and evolve with loading severity, strain rate, and material configuration. Under quasi-static or low-energy conditions, damage initiation is commonly matrix-dominated, starting with microcracking and local matrix yielding, followed by fibre–matrix interfacial debonding and the onset of localised delamination at interfaces where stress concentrations develop [83,127]. With an increase in the severity of the load, and especially under impact and other high-rate events, other failure modes come to the forefront, such as yarn splitting, localised fibre fracture, shear-driven failure, and widespread interlaminar delamination networks. Importantly, these mechanisms rarely occur in isolation, and intralaminar cracking and interfacial debonding can compete and initiate delamination growth, which in turn redistributes stresses and promotes intralaminar failure [128,129]. This qualitative evolution from matrix-dominated damage to delamination-dominated growth and finally to fibre-dominated failure is schematically shown in Figure 13.

**Figure 14:**
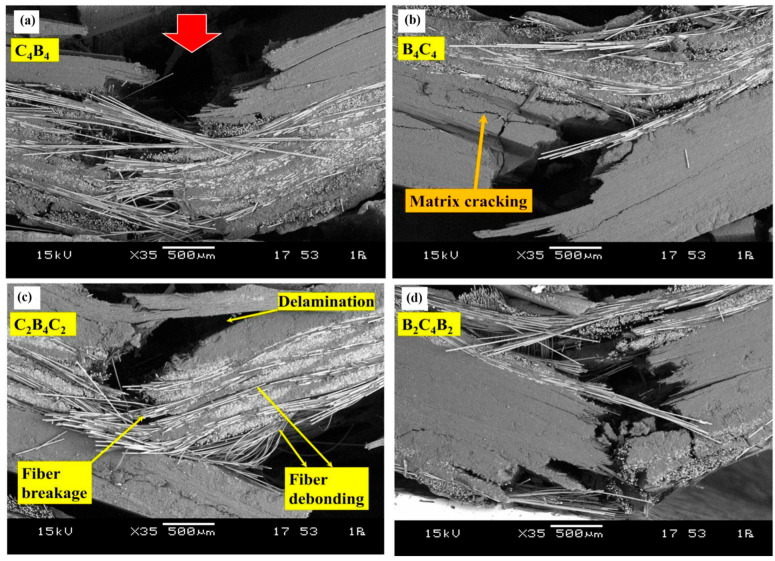
Representative fracture morphologies of hybrid carbon/basalt laminates after Charpy impact tests: (**a**) [C4B4], (**b**) [B4C4], (**c**) [C2B4C2], and (**d**) [B2C4B2], showing matrix cracking, delamination, fibre breakage, and fibre debonding/pull-out (scale bars as in the original). C = carbon-fibre ply; B = basalt-fibre ply; subscripts indicate the number of consecutive plies. Adapted from [82].

Fracture morphology gives vital insight into the mechanism of the interaction among fibres, matrix, and woven architecture. In hybrid systems, fracture surfaces frequently show mixed-mode features, reflecting the coexistence of stiff/brittle and ductile constituents and the associated competition between catastrophic and progressive failure pathways [133,134]. Typical observations are brittle breakage of fibres, ductile pull-out and fibrillation of fibres, tearing of the matrix, and interfacial separation, the relative strength of which depends on the fibre type, surface chemistry, interface quality, yarn crimp, and the hybrid fibre distribution [69]. Fibre pull-out and interfacial debonding are particularly influential because they control stress transfer and energy dissipation during crack propagation; consequently, they are often associated with more gradual strength degradation and improved damage tolerance compared with monolithic systems. Figure 14 illustrates the representative pull-out and interfacial damage features spanning single-fibre and fibre-bundle scales.

#### 4.4.3. Non-Destructive Testing Techniques

BVID is a significant issue in the assessment of the structural integrity of HPWCs since the damage caused by impact is largely localised in the subsurface and cannot be precisely identified by visual inspection alone. Non-destructive testing (NDT) techniques are fundamental for the detection of internal damage features as well as the quantification of their extent without compromising structural integrity [135,136].

Among the available techniques, ultrasonic testing is the most widely adopted and mature NDT technique for hybrid woven composites. Ultrasonic C-scan inspection has proven to be very sensitive for identifying delamination, voids, and interfacial debonding resulting from impact events. Several studies have shown that ultrasonic techniques provide an opportunity to accurately map the morphology of impact-induced damage and to quantitatively evaluate the damage of different laminate configurations [115,122,136,137,138]. However, ultrasonic interpretation is sometimes influenced by complex woven architecture (undulation/crimp of the yarns) and hybridization, and this can enhance scattering and attenuation as well as complicate the size and depth interpretation of defects [135,139]. Recent work has therefore been focused on the combination of ultrasonic measurements with complementary modalities (e.g., computed tomography) in order to minimise sizing bias and increase the confidence level in the estimation of the extent of the damage [136].

Infrared thermography has complementary capabilities to provide rapid and full-field inspection capabilities for surface and near-surface damage. Active thermography methods (e.g., pulsed/lock-in variants) have been applied for rapid detection and indicative sizing of impact-related damage in aerospace-grade composites, with the beneficial practicality of non-contact inspection and fast scanning [138,140,141]. However, thermography typically has a limited penetration depth in thick and highly heterogeneous laminates, which can restrict sensitivity to deeply embedded damage and complex through-thickness delamination networks [135,138,142].

X-ray computed tomography (CT) facilitates the visualisation of the internal damage and microstructural characteristics and can be regarded as a high-fidelity reference method of evaluating complex internal damage (e.g., delamination networks, intralaminar cracks, and architecture-related damage paths) [115,116,136,142]. In the case of woven and textile composites in particular, micro-CT studies thus underline the importance of 3D imaging both for the interpretation of the damage in terms of architecture, and for the practical limitations of costs, scan times, and specimen size, which may limit routine studies to large components [143].

Comparative studies always revealed that none of the NDT techniques are enough to fully characterise BVID in HWPCs, but instead, multi-modal strategies lead to better reliability. A practical combined approach is to use, for damage quantification, ultrasonic inspection for an efficient quantification of damage, thermography for a rapid screening, and selective X-ray CT for a detailed 3D damage analysis and to model validation-grade datasets [135,136,138,143]. Such combined techniques are also starting to keep up with the current trends towards data-rich validation and automation (including AI-assisted interpretation) discussed in recent NDT reviews for composite structures [130,135,144]. A comparative summary of the capabilities and practical suitability of the major NDE techniques for damage characterisation and model validation in HPWCs is provided in Table 5.

#### 4.4.4. Strategies to Improve Damage Tolerance

Improving the damage tolerance and residual strength after impact in HPWCs requires the integrated design strategy of (i) reinforcing architecture design, (ii) hybrid fibre selection and placement design, and (iii) matrix/interface engineering design, since these factors play a major role in controlling damage initiation, crack deflection/arrest, delamination growth, and energy dissipation under impact loading [23,108].

From an architectural standpoint, increasing the thickness of the reinforcement is one of the most effective routes to contain damage. Multiple studies and reviews report that 3D woven architectures (e.g., orthogonal and angle-interlocking architectures with binder/through-thickness yarns) are able to suppress or strongly limit delamination growth compared to the behaviour of 2D laminates, mostly due to the provision of through-thickness bridging and enhanced interlaminar fracture resistance [23,90]. In addition, in the case of woven structures without corresponding reinforcement of thickness, weave topology and interlacing constraints play a role in terms of impact response and damage distribution; comparative studies done on woven architectures have proven that tighter interlacing can cause changes in the crack path and energy dissipation by changing the mobility of the dissipation of frictional energy [146]. These observations support architecture-driven approaches that target delamination resistance and damage diffusion as primary design objectives in impact-critical applications [23,90].

Hybrid fibre architectures represent an added mechanism to improve the structure’s damage tolerance of the reinforced structure by using stiff and load-bearing fibres with more compliant and energy-absorbing reinforcements. In 3D orthogonal woven systems, intralaminar carbon/glass hybridisation has been reported to lead to increased strain to failure and energy absorption capacity with LVI by causing a more progressive evolution of damage than in non-hybrids [91]. Similarly, the hybrid woven laminates (e.g., carbon/aramid systems) under repeated low-velocity impacts show damage evolution, including matrix cracking, delamination, and fibre fracture, where the hybrid configuration and fibre orientations affect the rates of damage growth and post-impact integrity [147]. Collectively, the literature suggests that hybridization can be used to enhance damage tolerance through the redistribution of stresses at the yarn/laminate scale, through the delayed growth of unstable cracks and/or through more gradual failure; however, the extent of improvement is strongly dependent on the selection of constituents, volume fraction, and spatial arrangement within the woven architecture [91,147].

Matrix and interface engineering is also a key factor in the control of the initiation and propagation of impact damage. Comparative studies demonstrate that thermoplastic matrices can have higher impact resistance than thermosets in textile/fabric composites, which are usually attributed to higher matrix ductility, tougher interfaces, and higher interlaminar fracture toughness, which can reduce the damage area and retard the growth of damage under LVI [108,148]. Thermoset matrices (e.g., epoxy) provide high stiffness and strength but are intrinsically more brittle; therefore, matrix toughening is widely used to improve crack resistance and delamination performance. Reviews about epoxy toughening point out that rubber modification (including core shell rubbers), thermoplastic modifiers, and nanoparticle additions can improve the fracture toughness by the mechanism of particle cavitation, shear yielding, crack deflection/pinning, and increased energy dissipation [149,150,151]. In parallel, interface tailoring through fibre surface treatment and sizing has been reported to increase interfacial shear strength and, in some cases, reduce damage area by improving load transfer, though it may, in some cases, promote premature debonding [152,153].

In general, HPWCs with enhanced damage tolerance and post-impact performance can be achieved by the synergy of (i) architectures that limit the growth of delamination (including 3D woven designs where appropriate), (ii) hybrid fibre systems that favour progressive damage and energy absorption, and (iii) matrix/interface engineering that increases crack growth resistance and stabilises load transfer. These integrated strategies are part of the reliable performance of service and qualify in impact-sensitive, safety-critical applications [23,108].

## 5. Numerical Modelling and Finite Element Analysis of HPWCs

Numerical modelling, including finite element analysis (FEA), plays a critical role in understanding, predicting, and optimising the mechanical behaviour of HPWCs. The natural heterogeneity of woven architectures, hybrid fibre systems, and complicated damage mechanisms makes experimental characterisation, in many cases, not a sufficient technique to determine the complete spectrum of structural response to both steady and dynamic loading conditions. This has made computational methods essential to the mapping of material design parameters to macroscopic performance to allow parametric studies, virtual testing, and performance prediction in real-world conditions [13,29,36,41].

Within the framework of HPWCs, numerical modelling approaches will cover a wide range of length scales, including macroscopic representations that are homogenised and fibre-level descriptions. The modelling scales have their own strengths and weaknesses with regard to the accuracy, computational power, and applicability to engineering problems. The choice of a suitable modelling method, thus, relies on the desired response, loading regime, and the level of detail needed, especially in the case of impact behaviour, damage development, and residual strength. This section discusses the main modelling techniques employed in the various length scales, with a specific focus on their applicability to hybrid woven composite systems.

In practical terms, macroscale models are most appropriate for early structural sizing, global crash or impact-response prediction, and component-level parametric studies where computational efficiency is essential [41,42,83,153,154]. Their main limitation is that homogenisation suppresses architecture-sensitive information such as yarn-level stress concentrations, local delamination morphology, and constituent-specific failure sequencing [155,156]. Mesoscale models are more suitable when weave topology, yarn interactions, and interlaminar damage strongly influence performance, whereas microscale models are best reserved for constituent/interface development and mechanistic parameter identification [21,43,157,158,159,160,161,162]. For industrially relevant simulations, multiscale strategies are therefore often required, allowing local high-fidelity modelling in critical regions while retaining global computational tractability elsewhere.

### 5.1. Modelling Approaches Across Length Scales

#### 5.1.1. Macroscale Modelling

At the macroscale, HPWCs are commonly represented as homogeneous, equivalent continua, commonly known as black-box models. In this method, the composite structure is modelled as a single material that has effective elastic, strength, and failure behaviour, without explicitly resolving the underlying fibre architecture, yarn interactions, or matrix distribution. This simplification allows the application of these models to large-scale structural studies where computational efficiency and prediction are prioritised over detailed damage resolution [41,42].

In macroscale models, the successful characterisation of material properties is typically based on either experimental characterisation, analytical homogenisation, or lower-scale numerical simulation. Elastic parameters, strength parameters, and failure parameters are also set to reflect the average response of the hybrid woven composite at certain loading conditions. As reported by Chen D et al. [83], homogenised models can accurately forecast the global stiffness, load displacement behaviour, and the total failure loads precisely when the material response is still dominated by elastic deformation and the distributed damage. The modelling approach remains extremely attractive in the preliminary design, structural optimisation, or certification level simulation engineering problems due to its ease and robustness.

A primary advantage of macroscale modelling lies in its low computational cost, which allows for the simulation of complex components and full-scale structures subjected to realistic boundary conditions and loading scenarios. This makes homogenised models adapted well to impact simulations at the structural level, crash analysis, and parametric studies in which a number of design variables are varied [154,163]. Moreover, they are compatible with commercial finite element software, making them easy to adopt in an industrial environment.

However, the main drawback of macroscale modelling is that it does not explicitly define the damage initiation and progression processes that are inherent to hybrid woven composites. Local phenomena like matrix cracking, fibre–matrix debonding, yarn pull-out, delamination, and hybrid specific load redistribution cannot be directly solved within this framework. Therefore, predictive precision can be compromised when complex damage modes, strain rate effects, or post-impact residual strength are of interest [155,156]. Although the models of phenomenological damage and continuum damage mechanisms are commonly introduced to address these limitations, their parameters are highly reliant on the experimental calibration process and might not apply to other hybrid architectures and loading regimes [122].

In general, homogenised macroscale modelling is an efficient and practical instrument of analysis of the global structure of HPWCs. Nevertheless, it is limited by its inherent simplifications, which restrict its use in predicting damage in detail and microstructural interpretation, which drives the creation and implementation of mesoscale and multiscale methods, as discussed in the following sections.

#### 5.1.2. Mesoscale Modelling

Mesoscale modelling provides an intermediate-level description in which the internal structure of HPWCs is explicitly determined at the laminate, ply, or yarn levels. Unlike homogenised macroscale approaches, mesoscale models represent the spatial distribution of reinforcement layers, the interlacing pattern between the yarns, and the rich areas of the matrix with a more physically significant representation of the initiation and propagation of damage during mechanical loading. This modelling has gained a wide usage in the exploration of failure mechanisms of woven and hybrid composite systems [43,69].

In ply-based mesoscale frameworks, the composite laminate is resolved into separate layers that have different material characteristics and constitutive laws. The method is especially efficient in the case of hybrid laminates based on various types of fibres, when the difference in the stiffness, strength, and strain to failure of the plies has a profound effect on the response on the global scale and damage development. Damghani M et al. [158] found that the stiffness degradation and progressive failure of hybrid laminates can be realistically modelled at the ply level. Nevertheless, the effectiveness of this method depends on proper interlaminar modelling that should be used to describe delamination and inter-ply damage since these two processes are essential in the overall failure process [128].

In the case of woven composites, a more detailed description is provided by mesoscale modelling at the yarn level, which explicitly models the warp and weft yarns, their crimp, and how these interact with the polymer matrix. This approach enables the local stress concentration at the crossover points of the yarns to be analysed and the effects of hybridisation at the architectural level to be studied. In particular, it enables the exploration of the interaction of stiff and ductile yarns in a woven fabric and can provide more information on load redistribution, damage location, and the sequence of failures in different loading conditions (tensile, compressive, and impact loading) [21]. As observed by Yang N et al. [162], yarn-based models provide important information on damage propagation mechanisms and the contribution of hybridization to improve the performance of composite materials under dynamic loading conditions.

Mesoscale finite element frameworks are based on layer-wise damage and delamination modelling. Damage initiation is commonly formulated in terms of stress or strain criteria, whereas damage evolution is formulated in terms of continuum damage mechanics or cohesive zone formulations. Cohesive elements or surface-based cohesive interactions have been commonly used to capture interlaminar delamination, a failure mode of critical importance in hybrid woven composites. The study by Ferreira L.M et al. [160] emphasises that the mesoscale models with the inclusion of cohesive interfaces can be used to successfully model experimentally observed delamination patterns and stiffness degradation under dynamic loading.

The main benefit of mesoscale modelling is that it can model the progression of failures at the laminate and yarn scales, and thus directly connects the material architecture and macroscopic behaviour. This capability is particularly important, as it enables the evaluation of hybrid-specific damage mechanisms that cannot be resolved using homogenised approaches. They also provide better predictive capability of impact damage, delamination growth, and residual strength than macroscale models, especially when backed up by experimental calibration and validation [157].

However, mesoscale modelling also has a number of limitations. The explicit modelling of layers and yarns makes it much more expensive to compute, and not possible in large structural partial or full-scale simulations. In addition to this, modelling complexity and uncertainty are presented by the definition of boundary conditions, contact interactions, and interfacial properties. To guarantee a numerically tractable model, assumptions about perfect bonding, reduced yarn geometry, or homogeneous material properties are frequently necessary, diminishing the quality of predictions [164]. Moreover, the parameterization of materials needed in hybrid systems is a challenge in terms of model transferability between different composite structures and loading conditions [155].

In general, mesoscale modelling based on layer- and yarn-level modelling can provide a strong trade-off between accuracy and computational efficiency to mechanistically elucidate the damage development and hybrid behaviour in polymeric woven composites. Although mesoscale modelling has computational requirements and the complexity of defining boundary conditions, it is still critical to understanding the failure mechanisms at the laminate and yarn levels.

#### 5.1.3. Microscale Modelling

Microscale modelling is the most detailed level of numerical representation for HPWCs, with a solution that includes the individual fibres, the polymer matrix, and their interfaces being explicitly resolved. This approach is necessary to capture the basic mechanisms of the damage initiation, transfer of the load, and interfacial failure that govern the macroscopic response of composite systems. In recent studies [43,161,165,166], microscale models have been used increasingly to study the role of fibre–matrix interactions in hybrid composites, which consist of fibres of dissimilar mechanical properties that are present in the same matrix, such as carbon, glass, aramid, and natural fibres.

The main framework for microscale analysis is based on Representative Volume Element (RVE) modelling. In this method, a statistically representative part of the composite microstructure is made and placed under boundary conditions according to the macroscopic loading state. RVE-based simulations are able to support the explicit representation of fibre geometry, spatial distribution, and interfacial characteristics that allow detailed stress and strain field analysis at the constituent level. As reported by Balasubramani N.K. et al. [167], such models are especially effective at capturing the matrix cracking, fibre breakage, and interfacial debonding, which are often precursors to damage evolution on larger length scales.

For the case of hybrid woven composites, the use of microscale RVE models allows for unique information in the interaction between various types of fibres, such as carbon, glass, aramid, and natural fibres embedded in a polymer matrix, typically consisting of a mixture of these polymers. The present study by Nejad et al. [159] emphasises that microscale simulations can be used to identify localised stress concentrations caused by stiffness mismatches between fibres, as well as hybrid-specific damage initiation patterns that cannot be resolved using homogenised or mesoscale approaches. The ability to deform and optimise interfacial strength dependently on strain rate, as well as the impact of matrix properties on damage initiation, is especially important for microscale modelling [159].

The greatest advantage of microscale modelling is that it is highly predictive. The explicit derivation of the constituent-level mechanisms of these models gives a physically motivated basis on which simulations can be conducted in the mesoscale ranges and macroscale ranges by calibration and development of constitutive laws. In addition, microscale studies may be applied to material design so that virtual testing of fibre surface treatments, matrix modifications, and hybrid arrangements can be performed before experimental validation [168].

**Figure 15:**
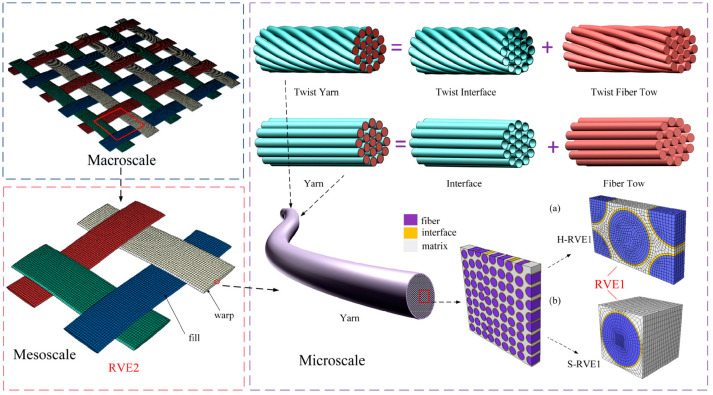
Multiscale modelling framework for 2D woven composites. Reproduced from ref. [169].

However, microscale modelling is computationally intensive and typically limited to small domains and simplified loading conditions. Its accuracy depends strongly on the availability of high-resolution material data, including fibre geometry and interfacial properties, which are often difficult to obtain experimentally. As a result, the direct use of microscale models for full-scale structural analysis is still impractical, and their use is often limited to the parametric study of physical quantities or as the input of multiscale frameworks. A representative example of such a microscale-to-macroscale linkage is illustrated in Figure 15, which summarises a multiscale modelling workflow for 2D woven composites considering the twisted angle and interface in RVE. The H-RVE is fibre arrayed in a hexagonal pattern, and the S-RVE is fibre arrayed in a square.

#### 5.1.4. Homogenisation and Multiscale Modelling Frameworks

Homogenisation and multiscale modelling give a systematic method of resolving the various length scales in the numerical simulation of HPWCs. Owing to the pronounced heterogeneity introduced by fibre–matrix interactions, woven structures, and hybrid reinforcement strategies, single-scale models will commonly not be able to predict mechanical behaviour with the necessary accuracy over a wide range of loading regimes. The objectives of multiscale strategies are to relate the microscale processes of damage to the mesoscale architectural impact and ultimately the macroscale structural reaction, which are important issues concerning the scale-dependent material performance [43].

The multiscale modelling is based on scale transition strategies, which may be generally divided into hierarchical and concurrent strategies. In hierarchical models, lower-scale simulations are used to obtain effective properties and constitutive parameters, such as microscale RVE simulations, and then transfer these parameters to mesoscale or macroscale models. Although He C et al. [43] have found hierarchical homogenization to be a promising tool that allows one to efficiently simulate large composite structures, its predictive abilities are highly dependent on the quality and the representativeness of the lower-scale models and the assumptions that are made during the process of scale averaging [170]. In contrast, multiscale methods are also concurrent, with multiple scales interacting in a unified simulation framework, so that localised high-fidelity modelling is done in regions of interest, but global computational efficiency is ensured elsewhere [60,171].

A central challenge in multiscale modelling is the compromise between computational efficiency and predictive accuracy. High-resolution microscale models can give a detailed understanding of fibre interactions, interfacial debonding, and matrix cracking, but are computationally expensive for large domains. On the other hand, macroscale homogenised models are efficient but can fail to capture important local processes that can control the initiation of damage and post-impact response [43,60]. As observed by Shi Jianwei et al. [172], hybrid multiscale frameworks seek to trade these competing demands by arbitrarily addressing local heterogeneity where it is needed with homogenised descriptions in less important areas.

Homogenisation and multiscale techniques are especially relevant to hybrid woven composites, where scale-dependent effects have a strong impact on material performance. The presence of mismatched fibres in terms of their stiffness, strength, and failure behaviour brings about intricate interactions that are varied at the fibre, yarn, and laminate levels. The article by Simon H et al. [173] highlights the fact that multiscale models can represent hybrid specific redistribution of loads and damage evolution patterns that cannot be solved with single-scale models [173,174]. These are necessary capabilities that help in the study of strain rate sensitivity, propagation of impact damage, and residual strength in hybrid systems.

Hybrid modelling approaches that use a combination of various scales in the same framework have become a new prospect towards dealing with the complexity of woven composite behaviour. These strategies combine microscale RVE-based damage models with mesoscale yarn-scale descriptions and macroscale structural analysis, which facilitate a complete evaluation of mechanical performance on both length scales. More recently, these hybrid models have been successfully used to model local fibre failure, yarn pull-out, and delamination at manageable computational costs [172].

Multiscale and homogenisation frameworks have some issues despite their strengths. The control of macro and heterogeneous collections of data on scales, the establishment of uniform border conditions, and the determination of scale-transfer factors also remain a challenging issue [170]. Moreover, getting numerical stability and convergence of coupled multiscale simulations means that models should be carefully designed and validated. By tackling those problems, it is necessary to enhance the strength and industrial usability of multiscale modelling methods with HPWCs.

#### 5.1.5. Constitutive Modelling and Parameter Calibration

One principal aspect of numerical modelling of HPWCs is constitutive modelling because it establishes the connection between stress, strain, evolution of damage, and failure under varying loading. As composite materials are heterogeneous materials in nature, particularly in hybrid structures, and because interactions between fibres, matrices, and interfaces are intricate, constitutive models that are both reliable and consistent to work with are a significant challenge in composite mechanics. Continuum damage mechanics (CDM) models have thus found extensive application to simulate progressive damage and stiffness degradation of hybrid composites under both dynamic and static loading conditions [122].

Damage initiation in CDM-based models is often stated in terms of stress or strain, while damage evolution equations are used to describe the progressive damage of materials. In the case of HPWCs, it is possible to expand these models to consider many failure modes such as fibre rupture, matrix cracking, and interfacial debonding. Chen Jing Pham D C et al. [175] showed that the formulations of CDM are capable of correctly modelling progressive failure in hybrid laminates, provided the damage variables and interaction terms that characterise hybrid-specific failure mechanisms are defined appropriately [176].

Constitutive formulations that are rate-dependent are required to model the behaviour of HPWCs during impact and high-speed loading. Polymer matrix fibres and fibre–matrix interfaces tend to be strain rate-sensitive and are usually viscoelastic or viscoplastic. According to Herguet et al. [177], rate dependencies in the material parameters improve the dynamic response, energy dissipation, and post-impact residual strength prediction. In hybrid systems, the strain rate sensitivities of the constituent fibres are different, which makes the hybrid more complicated and necessitates more sophisticated hybrid-sensitive parameterization strategies [178].

The constitutive parameters are also important in the predictability of the numerical models, and this can only be achieved through experimental calibration. The material properties are normally determined by a combination of quasi-static, dynamic, and impact tests, such as tensile, compressive, shear, and interlaminar fracture tests [32]. Multi-axis and rate-dependent data were emphasised in the research by Rehra et al. [69] as essential for fully describing the behaviour of hybrid composites, especially in the process of modelling the damage formation and failure of multi-complex loading. Practically, reverse modelling techniques are becoming common in order to reduce the differences between experimental measurements and numerical forecasting [179].

Even with tremendous progress, there are still problems with the experimental calibration and validation of constitutive models of hybrid woven composites. The calibration parameters can be subject to uncertainty due to experimental scatter, the inability to separate individual damage mechanisms, and the lack of data at high strain rates. Furthermore, constitutive models are not very transferable to other hybrid architectures and loading regimes, and have to be recalibrated with each new material system [29,71]. These are important problems that must be addressed to enhance the strength, generality, and industrial relevance of constitutive models for finite element simulations of HPWCs. Table 6 provides a summary of the constitutive and damage modelling approaches used in numerical simulations of HPWCs, the damage modes involved, the dependence on the rate, the calibration approaches, and the main benefits and drawbacks of these models.

### 5.2. FEA for the Prediction of Impacts and Damage

FEA-based impact and damage simulations are widely used to predict the dynamic response of HPWCs under high-strain-rate loading conditions. Applications dealing with the impact, collision, and ballistic phenomena require numerically based simulation frameworks that are able to take into account complex damage mechanisms, strain rate sensitivity, and energy dissipation processes, which are often hard to resolve experimentally. Consequently, FEA-based impact modelling plays a central role in virtual testing, structural optimisation, and failure evaluation of hybrid composite systems [96,183].

To simulate damage initiation, evolution, and final failure in HPWCs subjected to dynamic loading, progressive failure analysis (PFA) frameworks are commonly adopted. These approaches usually combine continuum damage mechanics (CDMs) for intralaminar degradation with cohesive zone models (CZMs) for delamination, implemented in explicit or implicit finite element solvers [184]. Recent studies about woven and laminated composites have shown that the woven and laminated frameworks are able to reproduce key experimentally observed mechanisms, i.e., matrix cracking, fibre rupture, delamination growth, interface-related damage, etc., provided appropriate failure criteria, characteristic length regularisation, and damage evolution laws are used and calibrated [184,185,186]. For hybrid woven systems, modelling needs to further model the unique constitutive response of the constituent fibres and their interactions in the woven architecture (e.g., stiffness mismatch, contact/friction, and hybrid-sensitive failure sequencing). While the use of hybrid-sensitive modelling strategies and architecture-resolved approaches can enhance the predictive fidelity, the coupling of multiple interacting failure modes in a numerically stable and computationally efficient manner is an ongoing challenge [30,185]. Nevertheless, progressive simulations offer valuable insight into load redistribution and damage-containment mechanisms controlling the resistance to impacts and the post-impact residual strength in HPWCs [30,187].

Impact simulations of HPWCs are most frequently carried out with explicit dynamic finite element solvers, which are well-suited for dealing with large deformations, nonlinear contact, and rapidly evolving damage. These simulations have been used for a wide variety of high-strain-rate events such as low-velocity impact, high-velocity impact, and ballistic penetration events. Numerous studies [28,93,94,96,99,159] emphasise that the use of strain rate-dependent constitutive behaviour is necessary to correctly predict stiffness, strength, and failure characteristics under dynamic loading conditions. Strain rate effects are mainly caused by the viscoelastic and viscoplastic responses of polymer matrices and interfacial regions, whereas for hybrid systems, the effects are more complex because of the different rate sensitivities of the constituent fibres. Hu X et al. [188] stated that neglecting the strain rate dependence may cause a large underestimation of the energy absorption and damage resistance in impact, which shows the importance of rate-dependent constitutive laws and damage evolution models in impact simulations of hybrid woven composites.

For predictive impact simulation, the most important experimentally constrained variables are typically: (i) intralaminar damage initiation and evolution parameters for fibre and matrix failure; (ii) cohesive traction-separation laws governing interlaminar delamination and interface debonding; (iii) rate-dependent viscoelastic or viscoplastic matrix/interface parameters; and, where relevant, (iv) contact and friction parameters associated with pull-out or sliding [177,183,184,185,188]. In this context, experimental quantities such as force–displacement histories, absorbed energy, damage extent and morphology, and ballistic-limit metrics such as V50 serve not only as descriptive measures of performance but also as calibration and validation targets for finite-element models [184,187,188,189]. This link is especially important in hybrid woven systems, where architecture-sensitive damage sequences and constituent mismatch can strongly influence both the measured response and the transferability of calibrated model parameters.

**Figure 16:**
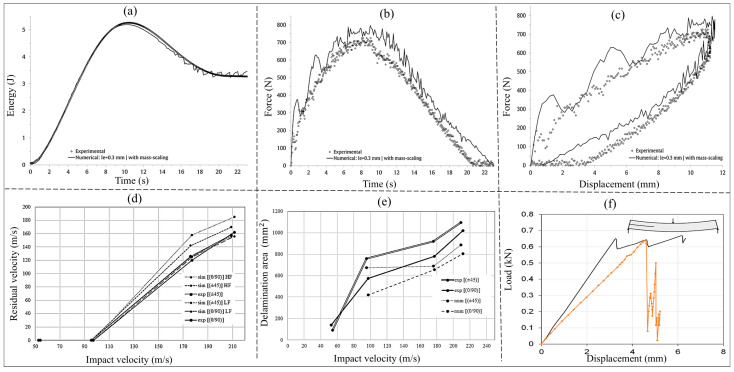
Experimental–numerical validation examples for FEA-based impact modelling of woven composite systems under LVI and HVI. (**a**–**c**) LVI: comparisons of experimental and simulated response histories for a woven composite shell, showing (**a**) energy vs. time, (**b**) force vs. time, and (**c**) force vs. displacement. Adapted from [35]. (**d**–**f**) HVI: experimental–numerical correlation examples for ballistic impact of woven FRP laminates, showing (**d**) residual velocity vs. impact velocity, (**e**) delamination area vs. impact velocity, and (**f**) load vs. displacement. Adapted from [190].

Experimental validation is an important part of FEM-based impact and damage prediction. Numerical results are usually verified by standardised experimental results of drop-weight impact tests, ballistic experiments, and instrumented impact tests. High-speed imaging techniques are frequently employed to acquire transient deformation, crack initiation, and crack propagation, where those data provide valuable benchmarks for numerical models [187]. Comparisons of simulated and experimental load time histories, force displacement responses, damage morphologies, and residual strength are often used to determine the fidelity of the model. As shown in Figure 16, experimental–numerical comparisons for woven composite systems under LVI and HVI have shown that FEA-based frameworks are able to capture key aspects of impact response, while showing areas where model refinement is still needed. While a number of studies show good qualitative agreement of simulations with experiments for hybrid woven composites [183], quantitative differences are frequently found due to material parameters, boundary conditions, and damage modelling assumptions. These observations underscore the need for rigorous calibration procedures and sensitivity analyses in impact modelling studies [183,189].

Recent advances in enhancing one’s predictive ability come in the form of improved progressive damage implementations (e.g., improved failure criteria and handling of characteristic length), better contact algorithms for impactor–structure interaction, and multiscale or selectively refined modelling strategies, which represent a good trade-off between fidelity and computational cost [184,185]. However, some issues remain in terms of capturing multiaxial stress states, mixed mode of delamination, and co-occurring damage interaction under extreme loading conditions, indicating that there is still development and validation of FE-based impact models for HPWCs [189].

### 5.3. Advances in Experimental Characterisation and Computational Modelling of HPWCs

Recent advances in HPWCs have been made possible by advances in both experimental characterisation and computational modelling to support an increasingly mechanism-informed assessment of mechanical response and damage evolution [23,191,192]. On the experimental side, there is a growing emphasis in the literature on richer datasets for model development and assessment—especially under dynamic and impact loading through better measurement strategies and multi-modal damage interrogation, including NDE for mapping the morphology of subsurface damage using ultrasonic inspection (e.g., C-scan), infrared thermography, and X-ray CT [136,145,193,194]. These modalities have been used to resolve impact-induced delamination’s and intralaminar damage with complementary sensitivity and spatial resolution that improve the link between measured damage patterns and numerical predictions [145].

A key development in simulation practice is the maturation of high-fidelity FE and multiscale approaches that take into account woven architecture, progressive damage, and constituent-level interactions in greater detail, thus allowing representation of failure processes in more detail than simplified ways [195,196,197]. Multiscale computational homogenization frameworks for textile/woven composites have been extended to complex 3D-textile architectures to support a more realistic prediction of effective behaviour with a retrained structured pathway of scale transition [198]. In parallel, multiscale progressive failure analyses have incorporated manufacturing-driven features (e.g., voids, disbonds, and tow waviness) and have demonstrated improved agreement with experimental response in representative 3D woven systems [195,196,199]. As a complement, FE^2^ (computational homogenization with nested FE problems) is also an important class of multiscale methods for composite mechanics and is still evolving with implementation and efficiency improvements, though it is computationally demanding for structural-scale problems [197,200].

Another major development is the rapid growth of artificial intelligence (AI) and data-driven modelling as complements to physics-based FE approaches. For woven and hybrid woven composites, deep learning surrogate models have been proposed to accelerate multiscale property prediction and enable fast exploration of architecture–property relationships [201,202]. Related advances also include deep learning frameworks for design optimisation of woven composites and neural network surrogates for reducing the cost of repeated lower-scale simulations in multiscale workflows [202]. More generally, neural network procedures have undergone review and application for constitutive modelling of composites, demonstrating the capacity to capture nonlinear and history-dependent behaviour given sufficient training data [203]. Beyond constitutive surrogates, graph-based machine learning approaches have been employed for accelerating field predictions in FE-type simulations, offering scalable pathways adaptable to composite simulation pipelines [204].

In the context of experimental data processing, deep learning methods are increasingly applied to automated damage detection, classification, and segmentation from CT and ultrasonic NDT datasets of woven composites, improving the consistency and efficiency of damage quantification [205]. Related AI-based approaches have also been applied to microscopic damage assessment of fibre-reinforced polymer composites using SEM images [206]. Bayesian inference and probabilistic calibration frameworks are also emerging as principled approaches for parameter identification in constitutive and damage models, enabling systematic quantification of model-form uncertainty and input variability challenges that are particularly acute in hybrid systems where constituent-level properties are rarely fully characterised [207,208]. Looking to the future, computational development is likely to place a greater emphasis on integrated, adaptive, and intelligent simulation frameworks (multiscale acceleration and surrogate-assisted workflows), along with digital twin concepts for structural health monitoring and lifecycle assessment, which are being actively reviewed and systematised in the wider SHM community.

Progress has also been made in the recognition and treatment of sources of uncertainty that cause discrepancies between experiments and simulations. Manufacturing-induced variability, including tow misalignment, resin-rich areas, and voids/disbonds, has been explicitly included in multiscale failure analyses of woven composites and has led to a better interpretation of the prediction scatter and failure sensitivity [195]. In parallel, stochastic multiscale homogenization approaches have been developed for woven textile composites in order to quantify the influence of constituent/property uncertainty on effective response [173,209]. More generally, uncertainty propagation and model form/statistical errors are becoming more common as fundamental components of multiscale composite simulation, which provides the motivation for probabilistic and reliability-oriented multiscale frameworks [210,211]. Looking to the future, computational development is likely to place a greater emphasis on integrated, adaptive, and intelligent simulation frameworks (multiscale acceleration and surrogate-assisted workflows) along with the digital twin concepts for structural health monitoring and lifecycle assessment, which are being actively reviewed and systematised in the wider SHM community [197].

## 6. Market Trends and Industrial Outlook

HPWCs are transitioning from specialised engineering solutions to mainstream industrial components, driven by application-specific performance requirements and sustainability mandates. This section examines how industrial demands shape materials development priorities, focusing on the relationship between application requirements and hybrid design strategies.

### 6.1. Global Market Overview

The global market for hybrid composites was valued at approximately $ 1.04 million in 2024 and is poised for continued growth [212]. This market is divided into application segments that have different drivers and material preferences, as summarised in Table 7. High-performance carbon/glass and carbon/aramid hybrids are used in the aerospace industry in high-performance structural parts where the strength-to-weight ratio and environmental durability are of primary importance [5,16,17,48,122,213]. The automotive industry, especially electric cars, uses glass/carbon and carbon/aramid composites to attain weight savings, crash-impact energy absorption, and control costs [48,73,214,215]. There are applications of wind energy that use carbon/glass turbine blades that need outstanding fatigue strength in cyclic loading and exposure to the environment [191,216]. The marine applications require corrosion-resistant materials, and the hulls and decks are made of the glass/carbon hybrids [217,218,219]. Construction is an emerging application field for hybrid FRP composites, particularly in structural strengthening systems, hybrid reinforcing bars for reinforced concrete, pultruded profiles, and cable elements for civil infrastructure, where low self-weight, corrosion resistance, and durability are important design advantages [9,37,48,49,220,221].

In the geographical market, North America dominates with a 50% share in 2024, driven by strong demand in aerospace, automotive, and construction, and supported by progressive regulations and key industry players like Hexcel Corporation and DuPont. Europe comes next with a market share of 25%, and the growth is driven by their strict environmental laws, especially in the automotive and aerospace sectors. The Asia-Pacific region is the most rapidly expanding with 16% of the world market, with high-paced industrialisation and governmental efforts in such countries as China and Japan driving adoption in the automotive industry and renewable energy. The Middle East and Africa region occupies a 9% share and is at the initial stages of adoption, with investments in construction and automotive being signs of transition to sustainable and lightweight manufacturing practices [212].

### 6.2. Forecast and Future Trends

**Figure 17:**
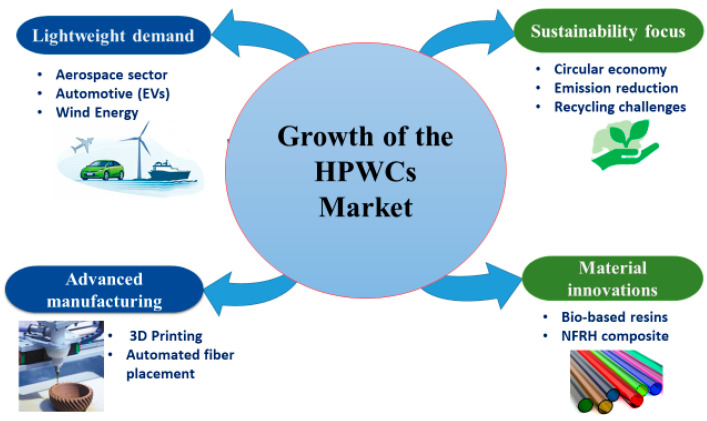
Converging trends shaping the HPWC market.

The HPWC market is expected to continue its growth, with projections estimating a market size of 2.05 USD million by 2035, reflecting a compound annual growth rate of 6.36% from 2025 to 2035 [212]. The factors that contribute to this growth include the rising need to use lightweight materials, the development of manufacturing technologies, and the rising sustainability concerns in various industries (Figure 17). The major industries, like aerospace, automotive, and construction, are still at the heart of this growth as these industries strive to achieve both performance and environmental objectives [224,225].

One of the key factors behind this increase is the need for lightweight materials in the aerospace and automobile industries, where hybrid composites can be used to satisfy the needs of fuel efficiency and emission reduction. Aerospace continues to lead due to the need for high strength-to-weight ratios and environmental stress resistance [16,213,220,226]. In the same manner, the increased use of hybrid composites in the automotive sector, particularly in electric vehicles (EVs), is the reason why vehicles become lighter and more energy efficient [37,214,215]. Wind energy is also a major participant in this market, especially in the development of turbine blades, where strength and fatigue resistance are of paramount importance [191,216,218,222].

Technological advancements in additive manufacturing (3D printing) and automated fibre placement are expected to accelerate market growth by enabling more efficient production of high-performance structures with reduced material waste [226]. Additionally, innovations such as thermoplastic composites [16], thermosets [227], and bio-based [27,51,84] resins are contributing to the development of sustainable materials, offering improved recyclability, performance, and reduced carbon footprints [27,37]. Moreover, there is an increasing trend in the incorporation of natural fibre-reinforced hybrids in the quest to find eco-friendly solutions without necessarily affecting mechanical performance [37,48].

Despite this promising outlook, significant challenges must be addressed to realise the full potential of HPWCs. These include the high production costs of advanced fibres and resins, the need for standardised lifecycle assessments (LCAs) to validate environmental benefits, and the critical development of efficient recycling pathways for end-of-life composite materials [220]. Overcoming these hurdles is essential to ensure the widespread and sustainable adoption of hybrid composites across industries.

## 7. Discussion

### 7.1. Architecture, Mechanical Performance, and Modelling Interplay

The evidence synthesised across Section 4 and Section 5 reveals a fundamental interdependence that is rarely made explicit in individual studies: reinforcement architecture does not merely govern mechanical response; it simultaneously defines the boundaries of what numerical models can and cannot predict with confidence. Architectural design decisions, from weave topology and hybridisation strategy to matrix selection, determine which damage mechanisms activate, at what scale they evolve, and how sensitive the resulting behaviour is to modelling assumptions. Recognising this three-way coupling between architecture, damage evolution, and simulation fidelity is therefore crucial for the development of both experimental understanding and predictive modelling of HPWCs. As synthesised in Table 2, Table 5 and Table 6, three practical trends emerge from the reviewed literature: hybrid architectures deliver their clearest advantages when progressive damage tolerance and stiffness–toughness balance are design priorities; the most suitable modelling scale depends strongly on the damage mechanism and required design fidelity; and industrial relevance increases when material selection is linked explicitly to application-specific loading and qualification needs.

A key insight from the reviewed literature is that the relationship between weave architecture and damage tolerance is not monotonic and cannot be reduced to simple classifications of “better” or “worse” architectures. Plain weave architectures, despite their lower in-plane stiffness, which is a direct consequence of higher yarn crimp, often demonstrate superior resistance to delamination propagation under impact loading. This is not coincidental: the same crimp that penalises stiffness can constrain crack growth through yarn interlocking and frictional energy dissipation. Twill and satin weaves, by reducing crimp, recover in-plane efficiency but also modify the local stress state and stress concentration landscape, which can shift the dominant failure mode from distributed matrix cracking toward more localised delamination under dynamic loading. 3D woven architectures address delamination through-thickness binder yarns but introduce their own modelling penalty: explicit representation of yarn interlocking, nesting, and binder warp interactions requires substantially more sophisticated and computationally expensive frameworks than 2D architectures demand. This architecture-specific modelling cost is a structural barrier to the routine use of high-fidelity simulation in 3D woven HPWC design.

Hybridisation adds a further layer of complexity that interacts with rather than simply overlays the architectural effects described above. Collective experimental evidence indicates that the mechanical benefits of hybridisation are highly configuration-sensitive: the same fibre combination (e.g., carbon/aramid) can have very different results depending on whether hybridisation is implemented at the interlayer, intralayer, or yarn level, and depending on the weave topology of each of the constituents. Critically, the hybrid effect on progressive failure is not simply additive. Carbon/glass and carbon/aramid systems often improve damage tolerance because the more ductile constituent delays unstable crack propagation, but the onset and degree of this delay depend strongly on stiffness mismatch, fibre volume fraction, and spatial arrangement variables that are rarely systematically controlled across studies. For natural/synthetic hybrid systems, the additional complications of moisture sensitivity and natural-fibre variability mean that performance scatter is intrinsic to the material class, not merely a product of imprecise manufacturing. These observations collectively mean that hybrid design optimisation cannot be based on single-variable experimental campaigns but needs to be based on integrated experimental–numerical frameworks that are capable of resolving architecture–hybrid–damage interactions simultaneously and separating true hybrid effects from scatter due to variability.

Under dynamic and impact loading, these interactions are enhanced. The reviewed evidence is consistent in showing that multi-modal damage in HPWCs, which includes matrix cracking, inter-yarn debonding, delamination, yarn splitting, fibre fracture, and fibre pull-out, does not occur in independent ways but in coupled architecture-dependent sequences. Residual strength after impact is particularly sensitive to this coupling: strategic placement of more ductile fibre components in outer layers or impact facing plies has been linked to reduced BVID severity and more distributed damage, but the mechanistic threshold at which hybridisation transitions from beneficial to neutral remains poorly understood. This gap is not only academic; it directly limits the ability of constitutive models to reliably predict post-impact behaviour without case-by-case recalibration and undermines model transferability across hybrid layouts.

Matrix selection is an additional design axis, whose effects on mechanical performance and on the complexity of modelling are underestimated in much of the reviewed literature. Thermoset matrices (e.g., epoxy, vinyl ester) provide high stiffness and thermal stability but tend to exhibit brittle fracture behaviour under impact, promoting rapid matrix cracking and delamination onset. Thermoplastic matrices provide greater toughness and rate-dependent deformation that can greatly enhance impact energy absorption and damage growth. However, accurately capturing these advantages in simulation requires viscoelastic or viscoplastic constitutive formulations coupled with rate-dependent damage and interface evolution capabilities that are not consistently implemented or calibrated in published frameworks, particularly in hybrid architectures where interfacial behaviour between dissimilar fibres and the matrix can dominate global response.

The relation between the complexity of architecture and modelling capability is best revealed in the scale-dependent accuracy of finite element approaches. Macroscale homogenised models are computationally efficient and well-suited to structural-level analysis, yet the evidence reviewed here consistently reveals that damage in HPWCs is spatially localised, architecture-dependent, and sensitive to constituent-level interactions that homogenised descriptions cannot resolve. As a result, these models often underestimate the extent of BVID, misrepresent the morphology of the delamination, and produce residual strength estimates that diverge from the experimental values for complex hybrid configurations, especially when the intent of the design depends on local mechanisms rather than only global stiffness and strength. Mesoscale models substantially improve predictive fidelity by explicitly representing yarn geometry, weave architecture, and inter-yarn contact; they correlate better with experimental impact force histories and damage patterns, particularly in hybrid systems where stiffness contrasts amplify local stress concentrations. However, their computational cost and sensitivity to interface/contact parameters, which are difficult to characterise experimentally, limit their practical use in large-scale parametric studies and design optimisation. Microscale models provide the highest mechanistic resolution and can support physically motivated parameter identification but remain confined to small representative volumes and simplified loading scenarios. Overall, the modelling lesson is scale-dependent: predictive capability increases with architectural resolution, but so do computational cost and the burden of calibration data, reinforcing the need for architecture-informed model selection and hybrid-sensitive parameterisation strategies.

Most validation studies in the literature compare global force–displacement response, absorbed energy, and residual strength with numerical predictions. Although qualitative agreement is commonly achieved, quantitative discrepancies in damage extent, morphology, and post-impact performance remain the norm rather than the exception. A key reason is the mismatch of the metric model: global curves may validate overall energy and stiffness trends, but do not confirm whether the correct damage mechanisms and sequences are captured, particularly in woven hybrids where architecture controls localisation. The use of more sophisticated experimental diagnostics, such as high-speed imaging, digital image correlation, and non-destructive multi-modal evaluation, has resulted in a finer spatial resolution of the available validation data and the ability to compare more than global curves. Nevertheless, consistent and standardised experimental-computational benchmarking strategies that are adapted to hybrid woven architectures are still missing. Consequently, although numerical models have reached a degree of maturity in the prediction of global structural response, reliable prediction of damage evolution and post-impact residual behaviour is an open challenge. Addressing this will require closer coupling of experiment and simulation, hybrid-specific and interface-sensitive damage formulations that include strain rate effects, and more rigorous and standardised validation protocols that are consistent with model resolution.

From a design perspective, the reviewed evidence indicates that HPWCs provide their most meaningful advantages over monolithic systems when local damage tolerance, progressive failure, or cost-constrained stiffness–toughness balance is more important than maximising absolute in-plane stiffness alone. Carbon/glass and carbon/aramid hybrids are therefore particularly attractive for lightweight structural panels, crash-relevant automotive components, impact-tolerant secondary aerospace structures, and protective shells where distributed damage and post-impact integrity are critical. In terms of architecture, 3D woven systems are preferable when through-thickness integrity and delamination suppression govern performance, whereas 2D twill and satin systems remain advantageous where in-plane efficiency, manufacturability, and lower modelling cost are dominant considerations. Model selection should follow the design questions: macroscale approaches are suitable for early structural sizing and global crash response; mesoscale models are required when architecture-sensitive delamination, yarn-level stress concentrations, or impact-damage morphology govern design; and microscale models are most appropriate for constituent and interface development, where their high computational cost can be justified by the need for mechanistic resolution. Overall, the principal challenge for the community is to convert these architecture-sensitive experimental findings into validated, transferable simulation workflows that can support optimisation, qualification, and industrial deployment.

### 7.2. Key Challenges, Research Gaps, and Priorities

Despite substantial progress in experimental characterisation and numerical simulation of HPWCs, several cross-cutting challenges still limit predictive reliability, inter-study comparability, and translation to design, optimisation, and qualification workflows. From a technology-development perspective, the most important community needs are architecture-sensitive test standardisation, transferable hybrid-specific constitutive and interface models, and validation procedures that remain credible under service-relevant loading conditions.

From the experimental point of view, one major drawback is the lack of standardised experimental test protocols specific to hybrid woven architecture. Although there are standards for conventional fibre-reinforced composites, they are not readily transferable to hybrid woven systems with dissimilar fibres and matrix behaviours, especially for impact and BVID assessment, where hybrid-sensitive interactions can control damage initiation, localization, and progression. As a result, impact/BVID setups, boundary conditions, and reporting practices differ between studies, compromising comparability between studies and the robustness of datasets used for model calibration and validation. A second major experimental limitation is the large amount of scatter that is often observed in strength and post-impact residual performance. This variability is because of the inherent heterogeneity of woven hybrids, non-uniform fibre distribution, architectural variability, defects introduced during manufacturing, and interfacial quality variation; thus, nominally identical hybrid specimens can even display strongly different damage development under similar loading conditions. Such scatter makes making sense out of it difficult and limits the reliability of parameter identification for models designed to predict localised damage measures, such as BVID extent and post-impact residual strength.

On the numerical side, correct modelling of interfacial behaviour and delamination is a major challenge. The existence of fibres with mismatched mechanical qualities and chemistries of the surfaces presents complicated interfacial interactions that are challenging to model with cohesive zone or continuum damage formulations without extensive, architecture-specific calibration. In many cases, key interface parameters are assumed or indirectly fitted, since direct experimental identification remains limited, reducing predictive confidence and model portability across hybrid configurations.

In addition, despite the fact that mesoscale and microscale models can capture hybrid-sensitive mechanisms in a more realistic manner, their computation cost and parameter sensitivity are limitations for their use in large-scale structural analyses, parametric analyses, and optimisation, reinforcing the dependence on efficient homogenised models that are often inadequate for the prediction of the evolution of fine-scale damage under dynamic and impact loading. Data scarcity further contributes to these problems; databases of material properties from hybrid systems are currently still limited, especially strain rate-dependent properties, interface and delamination parameters, and post-impact residual behaviour. Since most of the available datasets are based on single-fibre systems, many of them are not sufficient to describe the hybrid specific responses, hindering the generalisability of models and delaying the progress of developing transferable virtual testing and certification frameworks.

These limitations show research gaps and priorities that need to be addressed to make technology more ready for industry and to enable industrial adoption. First, impact and post-impact characterisation is incomplete: many studies focus on LVI of narrow values of energy and idealised boundary conditions, and comprehensive datasets in relation to damage morphology and residual strength are still limited for reliable structural reliability assessment. Second, long-term durability under environmental exposure is still underexplored. Moisture absorption, thermal cycling, and ultraviolet radiation may have a differential impact on constituent fibres and matrices in hybrid systems, which potentially leads to faster degradation of hybrid systems after incorporation of bio-based reinforcements, but systematic and service-relevant durability research is lacking. From a modelling perspective, there is a clear need for hybrid-specific constitutive and damage models that explicitly represent fibre-dependent damage evolution, interface- and delamination-driven mechanisms, and strain rate effects across length scales, so that virtual testing, design trade-offs, and qualification decisions can be made with traceable confidence. Finally, while HPWCs provide potential for lightweight and damage-tolerant structures for applications in the aerospace, automotive, and energy industries [20,27,29,37,38,48,49,66,82,92,213,220,222,228], wider deployment of HPWC materials is limited due to the cost of manufacturing, recyclability, and durability under service-relevant conditions [20,220,228,229,230], which must be resolved in order for them to be qualified in applications that have safety-related requirements. To consolidate the main evidence-based conclusions of this review and make the connection to the research questions explicit, Table 8 provides a compact RQ-linked synthesis of key findings, gaps, and priority research directions.

## 8. Conclusions and Future Perspectives

### 8.1. Conclusions

This review presents a critical synthesis based on a structured literature search on hybrid polymeric woven composites (HPWCs), with particular focus on mechanical performance, damage tolerance, and numerical modelling strategies. The main conclusions are as follows:

- Architecture is the central design variable in HPWCs. Weave topology, hybridisation configuration, and matrix/interface selection collectively govern load transfer, damage initiation, and residual strength after impact.
- Hybridisation can provide measurable mechanical benefits, but these benefits are highly configuration-dependent. Fibre placement, volume fraction, stacking sequence, and interfacial quality strongly influence the extent of the hybrid effect.
- BVID and post-impact residual strength are critical parameters for structural qualification, yet they remain difficult to predict consistently and are not yet supported by sufficiently standardised testing approaches.
- No single modelling scale is sufficient to describe HPWC behaviour comprehensively. Macroscale models are efficient for structural response, whereas mesoscale and microscale approaches provide better damage resolution, highlighting the importance of multiscale modelling strategies.
- Validation remains a major challenge. Agreement with global force–displacement or energy curves alone is insufficient, and greater emphasis is needed on mechanism-resolved benchmarking using spatially informed experimental data.

Overall, HPWCs show strong potential as damage-tolerant lightweight structural materials. Their broader industrial adoption depends on progress in experimental standardisation, improved understanding of hybrid-sensitive damage mechanisms, and the development of transferable, validation-driven modelling frameworks.

### 8.2. Future Perspectives

While this review has focused on fibre-scale hybridisation within woven polymer composite architectures, the synthesis highlights several directions that are likely to shape the next phase of HPWC development. Priority needs include: the establishment of HPWC-specific experimental protocols for impact, BVID, and post-impact residual strength, expanded multiaxial and high-strain-rate testing under service-relevant conditions; and the continued development of hybrid-sensitive modelling frameworks that combine physics-based multiscale approaches with data-driven acceleration.

Sustainability-oriented hybrid design will also become increasingly important, requiring the integration of bio-based fibres, recyclable matrices, and lifecycle assessment alongside rigorous durability evaluation. In parallel, emerging opportunities lie in smart and multifunctional HPWCs, including embedded sensing and structural health monitoring, to support validation-driven design and predictive maintenance.

Beyond fibre-scale hybridisation, nano-hybridisation using carbon nanomaterials (e.g., carbon nanotubes, graphene, and carbon nanofibres) represents a promising but distinct research direction. Such nanoscale reinforcements offer potential improvements in interfacial adhesion, damping behaviour, electrical conductivity, and fatigue resistance, and may enable hierarchical hybrid systems bridging nanoscales, mesoscales, and macroscales. A comprehensive treatment of nano-hybridisation, however, requires dedicated experimental methodologies and multiscale modelling frameworks and is therefore identified here as a priority avenue for future research rather than a core focus of the present review.

A consolidated overview of the key gaps, recommended actions, and expected impacts across these themes is provided in Table 8, which serves as the primary research roadmap derived from this review.

## Figures and Tables

**Table 1:** The research questions addressed in this review.

Research Question	Aim
RQ1. What are the important reinforcement architectures and matrix systems for HPWCs?	Clarify structural design and material choices.
RQ2. How does hybridisation of reinforcements affect mechanical behaviour and failure mechanisms?	Identify the role of hybrid reinforcement in enhancing performance.
RQ3. How do strain rate and dynamic loading affect hybrid woven composite properties?	Understand behaviour in impact and high-speed conditions.
RQ4. What is the residual strength of HPWCs in BVID?	Measure post-impact performance and damage tolerance.
RQ5. How is FEM used to simulate the behaviour of HPWCs and what are the limitations of this?	Evaluate modelling approaches and predictive accuracy.
RQ6. How does the inclusion of natural fibres affect sustainability and mechanical performance in HPWCs?	Evaluate sustainability–performance trade-offs.
RQ7. What are the publication trends and the industrial outlook for the HPWCs market?	Summarise the growth of research and industrial relevance.
RQ8. What are the most important barriers to the industrial scalability of HPWCs?	Assess barriers to large-scale manufacturing and adoption.
RQ9. What are the future research directions in mechanical testing, hybrid design, and simulation?	Provide guidance for future innovation and application.

**Table 2:** Review framework used to classify the literature on HPWs.

Classification Dimension	Categories Considered in This Review	Typical Examples Discussed
Hybrid type	Synthetic/synthetic	Carbon/glass; carbon/basalt; carbon/aramid
	Synthetic/natural	Glass/jute; carbon/flax; basalt/natural fibre
Reinforcement architecture	2D woven architectures	Plain, twill, and satin woven fabrics
	3D woven architectures	Interlock, orthogonal, and angle-interlock weaves
Matrix class	Thermoset matrices	Epoxy, vinyl ester, and polyester
	Thermoplastic matrices	Polypropylene (PP), and polyamide (PA)
Loading regime	Quasi-static loading	Tension, compression, flexure, and shear
	Rate-dependent loading	High-strain-rate mechanical response
	Impact and damage tolerance	Low-velocity impact, BVID, and post-impact residual strength
Modelling scale	Macroscale	Homogenised laminate-level FE models
	Mesoscale	Yarn-level and architecture-resolved models
	Microscale	Fibre–matrix interface and constituent-scale models
	Multiscale	Hierarchical, coupled, and FE^2^-type modelling frameworks

**Table 4:** Summary of BVID effects on residual strength of hybrid and weaved composite systems.

Composite System	Impact Energy Range (J)	Dominant BVID Characteristics	Reduction in Residual Strength (%)	Key Observations	Ref.
Carbon/epoxy (woven)	5–20	Matrix cracking, subsurface delamination	20–45	Significant loss in CAI strength despite minimal surface damage; delamination governs failure	[125]
Carbon/epoxy (woven, toughened by nano fibres)	5–20	BVID (permanent indentation < 300 µm)	30–50	Nanofibre interleaves improve impact damage resistance, and woven fabrics exhibit the highest improvements in CAI strength, particularly at 5 J (13%) and 22 J (26%)	[100]
Carbon/glass hybrid (woven)	10–30	Matrix cracking, distributed delamination	15–30	Glass fibres delay crack propagation and improve CAI compared to carbon-only fibres.	[66]
Carbon/aramid hybrid (woven)	15–40	Fibre pull-out, matrix cracking	10–25	Aramid improves energy dissipation and damage tolerance	[73]
Glass/epoxy (woven)	3–15	Matrix cracking, fibre–matrix debonding	15–35	Higher damage visibility but more gradual degradation	[126]
Flax/glass hybrid (woven)	10–25	Mixed fibre fracture, delamination	15–30	Hybridisation improves post-impact strength	[47]
Carbon/epoxy (multilayer laminate)	20–50	Extensive delamination	35–60	BVID leads to severe CAI degradation	[115]

**Table 5:** Comparative capacity and practical suitability of NDE techniques for damage characterisation and model validation in HPWCs.

NDE Technique	Primary Outputs	Capability by Damage Type (HPWCs)	Key Strengths (Why Used)	Main Limitations in HPWCs	Best Use for Simulation/Validation	Ref.
UT (pulse-echo, C-scan; phased array in some studies)	C-scan amp/TOF; B-scan; defect area	Delam H; Void M –H; Deb M; Wav M; µCrk L–M	Large area internal mapping; strong for interlaminar/BVID damage	Attenuation/scattering in woven/hybrids; depth sizing bias; calibration sensitive	Validate delam extent/morphology and BVID maps in impact models	[115,130,135,137]
IRT (pulsed/lock-in; + processing/ML in some studies)	Thermal contrast maps; defect screening	Delam (near-surf) M–H; Delam (deep) L–M; Void L–M; Deb M; Fibre frac L	Fast, full-field, non-contact screening	Limited penetration (thick/heterogeneous); emissivity/heating sensitivity; complex morphologies need processing	Rapid localisation + datasets for automated detection/segmentation	[135,138,140]
XCT/µCT	3D recon; delam network; void stats; architecture-resolved damage	Delam morph H; Void H; Deb M–H; µCrk M; Fibre frac M (resolution-dependent)	High-fidelity 3D “ground truth”; quantitative void/damage morphology	High cost/time; size limits; not for large structures	Validate detailed 3D damage morphology + defect statistics	[136,143,145]
Multi-modal (e.g., IRT→UT → XCT selective)	Complementary maps + selective 3D truth	BVID characterisation H (combined)	Combines speed (IRT) + mapping (UT) + 3D truth (XCT)	Coordination effort; no universal protocol	Benchmark workflows that link the extent of the damage ↔ morphology ↔ residual strength	[130,135,136,143]

Notes: H = high; M = medium; L = low; Delam = delamination; Void = voids/porosity; Deb = debonding; Wav = wrinkles/waviness; and µCrk = matrix microcracks.

**Table 6:** Constitutive and damage modelling frameworks implemented within numerical simulations of HPWCs.

Modelling Scale/Approach	Damage Modes Considered	Rate Dependence	Constitutive/Numerical Formulation	Calibration Methods	Key Advantages	Main Limitations	Ref.
Macroscale (homogenised/Black-box)	Global stiffness degradation, overall failure	Limited	Continuum damage mechanisms, phenomenological failure criteria	Quasi-static and impact tests, effective property fitting	Computationally efficient; suitable for large structures	Cannot resolve local damage mechanisms	[37,38,52]
Mesoscale (ply- and yarn-level)	Matrix cracking, delamination, yarn damage, fibre failure	Yes	Ply-level CDM, cohesive interfaces	Laminate tests, interlaminar fracture tests	Captures architecture-dependent damage and hybrid effects	High computational cost; complex parameter identification	[180,181]
Microscale (fibre–matrix/RVE-based modelling)	Fibre breakage, matrix cracking, and interfacial debonding	Yes	Constituent-level damage, cohesive interfaces	Micro-mechanical tests, RVE-based inverse calibration	High physical fidelity enables material design and mechanism insight	Computationally expensive; limited to small domains	[182]
Continuum damage mechanics (CDM)	Progressive damage, stiffness degradation	Yes (often coupled)	Damage evolution laws with stress/strain criteria; viscoelastic/viscoplastic extensions	Quasi-static, dynamic, and impact tests	Effective for progressive damage modelling; compatible with commercial FEM	Parameter sensitivity: recalibration required	[52,64,70]
Multiscale/homogenisation frameworks	Cross-scale damage evolution, load redistribution, and residual strength	Yes	Hierarchical or concurrent multiscale coupling	Multiscale experimental–numerical calibration	Balances accuracy and efficiency; hybrid-specific load redistribution	Complex implementation	[35,37,80]
Cohesive zone modelling (CZM)	Interlaminar delamination, interface debonding	Yes (rate-dependent traction-separation laws)	Traction-separation laws	Mode I/II/III fracture toughness tests; mixed-mode delamination	Accurate interface failure modelling	Limited to interfaces only	[64,70]

**Table 7:** Application-specific material requirements for HPWCs.

Application Sector	Market Share	Key Materials Used	Geographical Distribution	Primary Drivers	Ref.
Aerospace	Dominant	Carbon/glass, carbon/aramid, thermoset	North America, Europe, Asia-Pacific	Fuel efficiency, regulatory standards	[73,122,213]
Automotive	Significant	Glass/carbon, carbon/aramid, thermoset, thermoplastic	North America, Europe, Asia-Pacific	Light weighting, EV adoption, and emission standards	[214,215]
Wind Energy	Significant	Carbon/glass, thermoset	Europe, North America, Asia-Pacific	Renewable energy goals, turbine durability	[216,218,222]
Marine	Emerging	Glass/carbon	North America, Europe, Asia-Pacific	Corrosion resistance, marine environment challenges	[217,218]
Construction	Emerging	Glass/carbon, natural fibre hybrids	Global, growing in MEA & Asia-Pacific	Seismic retrofitting, structural integrity	[39,223]

**Table 8:** RQ-linked synthesis of critical gaps, recommended actions, and expected impact for HPWC research.

Area/RQ Focus	Synthesis Takeaway	Key Gap	Priority Research Action	Expected Impact	Linked RQ(s)	Refs.
Architecture and material systems	Mechanical response is governed primarily by weave architecture, hybridisation scale, and matrix/interface choice.	Lack of architecture-resolved, like-for-like comparisons across matrices and weave types.	Comparative datasets across 2D/3D architectures under harmonised test conditions.	Architecture-informed material selection and improved model calibration.	RQ1	[23,60,108]
Hybrid reinforcement and failure mechanisms	Hybridisation can enhance damage tolerance, but benefits are highly configuration-dependent.	Insufficient mechanistic understanding of fibre pairing and stacking effects.	Hybrid-specific studies linking fibre pairing, load redistribution, and failure sequencing.	More reliable hybrid design rules and controlled progressive failure.	RQ2	[58,69,73,85]
Strain rate & dynamic response	HPWCs exhibit strong rate sensitivity driven by matrix, interface, and fibre mismatch effects.	Limited high-rate and multiaxial datasets for hybrid woven systems.	Expanded high-rate and mixed-mode testing with rate-dependent characterisation.	Improved dynamic design envelopes and constitutive modelling.	RQ3	[93,94,98,177]
Impact, BVID & residual strength	Post-impact performance is architecture- and hybrid layout-dependent, but poorly standardised.	Sparse post-BVID residual-strength data and inconsistent damage metrics.	Standardised impact/BVID protocols linked to residual strength.	Safer qualification and improved damage tolerance prediction.	RQ4	[100,115,122]
Modelling & simulation	Models predict global response better than local hybrid-specific damage.	Limited fidelity and transferability of interface and delamination models.	Hybrid-sensitive multiscale and AI-assisted modelling frameworks.	Faster, higher-fidelity virtual design and assessment.	RQ5	[156,170,192,231]
Durability & sustainability	Natural/synthetic hybrids improve sustainability but raise durability concerns.	Limited long-term ageing and environment-coupled performance data.	Service-relevant ageing studies integrated with LCA and recyclability.	Improved service-life prediction and sustainability trade-offs.	RQ6	[44,47,51,87,232]
Industrial outlook & applications	HPWCs show growing relevance across sectors, but adoption pathways vary.	Weak linkage between hybrid design and sector-specific qualification needs.	Application-oriented benchmarking and qualification frameworks.	Clearer translation from research to industrial deployment.	RQ7	[6,218,220]
Industrial scalability	Adoption is limited more by cost, repeatability, and recycling than by proof-of-concept performance.	Limited manufacturing-scale and end-of-life evidence.	Scale-up studies, recycling routes, and techno-economic assessment.	Reduced barriers to large-scale manufacturing.	RQ8	[220,229,230]
Future directions & validation	Next advances require smarter validation and multifunctionality.	Lack of mechanism-resolved validation and real-time monitoring.	Smart composites, SHM-linked validation, and digital-twin workflows.	Higher model credibility and predictive maintenance capability.	RQ9	[120,130,197,233,234,235]

## Data Availability

No new data were created or analysed in this study. Data sharing is not applicable to this article.

## Supplementary Materials

The following supporting information can be downloaded at https://www.mdpi.com/article/10.3390/ma19091887/s1: Table S1: PRISMA 2020 Checklist.

## Abbreviations

The following abbreviations are used in this manuscript:

AI	Artificial intelligence
ASTM	American society for testing and materials
BVID	Barely visible impact damage
CAI	Compression after impact
CT	Computed tomography
CDM	Continuum damage mechanics
CFRP	Carbon fibre-reinforced polymer
CZM	Cohesive zone models
DIC	Digital image correlation
EV	Electric vehicle
FEA	Finite element analysis
FEM	Finite element method
FRPs	Fibre-reinforced polymers
HPWCs	Hybrid polymeric woven composites
HVI	High-velocity impact
IRT	Infrared thermography
ISO	International organisation for standardisation
LCAs	Lifecycle assessments
LVI	Low-velocity impact
NDT	Non-destructive testing
PFA	Progressive failure analysis
PRISMA	Preferred Reporting Items for Systematic Reviews and Meta-Analyses
RQ	Research question
RVE	Representative volume element
SCA	Self-consistent clustering analysis
SHM	Structural health monitoring
UD	Unidirectional
UHMWPE	Ultra-high-molecular-weight polyethylene
UT	Ultrasonic testing
XCT/µCT	X-ray computed tomography/micro-computed tomography
2D	Two-dimensional
3D	Three-dimensional
3DWIFs	3D warp interlock fabrics.

## Appendix A

**Table A1:** Annual publication counts (*n* = 2668; 2015–2025) [25].

Year	Documents	Share of Documents (%)
2025	344	12.9
2024	353	13.2
2023	284	10.6
2022	307	11.5
2021	249	9.3
2020	238	8.9
2019	246	9.2
2018	205	7.7
2017	166	6.2
2016	136	5.1
2015	140	5.2

**Table A2:** Top 20 countries/territories in the Scopus dataset (*n* = 2668; 2015–2025) [25].

Rank	Country/Territory	Documents	Share of Documents (%)
1	India	677	25.4
2	China	603	22.6
3	United States	222	8.3
4	Malaysia	183	6.9
5	United Kingdom	180	6.7
6	France	142	5.3
7	Turkey	102	3.8
8	Italy	93	3.5
9	Germany	88	3.3
10	Australia	84	3.1
11	Iran	69	2.6
12	South Korea	68	2.5
13	Saudi Arabia	66	2.5
14	Pakistan	59	2.2
15	Brazil	57	2.1
16	Japan	56	2.1
17	Belgium	49	1.8
18	Canada	47	1.8
19	Iraq	46	1.7
20	Egypt	40	1.5

Note: Country counts are based on documents with ≥1 affiliation in the country.

**Table A3:** Top 20 contributing affiliations/institutions in the Scopus dataset (*n* = 2668; 2015–2025) [25].

Rank	Affiliation/Institution	Documents	Share of Documents (%)
1	Ministry of Education of the People’s Republic of China	117	4.4
2	Universiti Putra Malaysia	67	2.5
3	Donghua University	66	2.5
4	CNRS Centre National de la Recherche Scientifique	50	1.9
5	Manipal Institute of Technology	33	1.2
6	King Mongkut’s University of Technology North Bangkok	32	1.2
7	Tiangong University	31	1.2
8	Manipal Academy of Higher Education	30	1.1
9	Harbin Institute of Technology	30	1.1
10	Saveetha Institute of Medical and Technical Sciences	29	1.1
11	Universiti Teknologi PETRONAS	26	1.0
12	The University of Manchester	26	1.0
13	Northwestern Polytechnical University	26	1.0
14	Universiti Teknologi MARA	26	1.0
15	KU Leuven	26	1.0
16	Saveetha School of Engineering	26	1.0
17	National Institute of Technology Rourkela	25	0.9
18	Chinese Academy of Sciences	25	0.9
19	Nanjing University of Aeronautics and Astronautics	25	0.9
20	Kalasalingam Academy of Research and Education	25	0.9

Note: Institutional counts are affiliation-based; a single document may be attributed to multiple institutions in multi-institution collaborations. Therefore, institutional shares do not necessarily sum to 100%.

**Table A4:** Top publication sources by journal name in the Scopus dataset (*n* = 2668; 2015–2025) [25].

Rank	Source Title	Documents	Share of Documents (%)
1	Composite Structures	123	4.6
2	Polymer Composites	44	1.6
3	Composites Part A: Applied Science and Manufacturing	36	1.3
4	Journal of Composite Materials	29	1.1
5	ICCM International Conferences on Composite Materials	29	1.1
6	Composites Science and Technology	28	1.0
7	Composites Part B: Engineering	24	0.9
8	Thin-Walled Structures	21	0.8
9	Applied Composite Materials	19	0.7
10	Fibers and Polymers	18	0.7

## References

[1] Risteska S., Srebrenkoska V., Zhezhova S., Srebrenkoska S., Risteski S., Jordeva S., Golomeova Longurova S. (2024). The Effect of Textile Structure Reinforcement on Polymer Composite Material Mechanical Behavior. Polymers.

[2] Codispoti R., Oliveira D.V., Olivito R.S., Lourenço P.B., Fangueiro R. (2015). Mechanical performance of natural fiber-reinforced composites for the strengthening of masonry. Compos. Part B Eng..

[3] Ramful R. (2024). Mechanical performance and durability attributes of biodegradable natural fibre-reinforced composites—A review. J. Mater. Sci. Mater. Eng..

[4] Mishra R.K. (2023). Advances in Textile Structural Composites. Polymers.

[5] Swolfs Y., Gorbatikh L., Verpoest I. (2014). Fibre hybridisation in polymer composites: A review. Compos. Part A Appl. Sci. Manuf..

[6] Swolfs Y., Verpoest I., Gorbatikh L. (2019). Recent advances in fibre-hybrid composites: Materials selection, opportunities and applications. Int. Mater. Rev..

[7] Sharma H., Kumar A., Rana S., Sahoo N.G., Jamil M., Kumar R., Sharma S., Li C., Kumar A., Eldin S.M. (2023). Critical review on advancements on the fiber-reinforced composites: Role of fiber/matrix modification on the performance of the fibrous composites. J. Mater. Res. Technol..

[8] Elanchezhian C., Ramnath B.V., Ramakrishnan G., Rajendrakumar M., Naveenkumar V., Saravanakumar M.K. (2018). Review on mechanical properties of natural fiber composites. Mater. Today Proc..

[9] Safri S.N.A., Sultan M.T.H., Jawaid M., Jayakrishna K. (2018). Impact behaviour of hybrid composites for structural applications: A review. Compos. Part B Eng..

[10] Chowdhury I.R., Summerscales J. (2024). Woven Fabrics for Composite Reinforcement: A Review. J. Compos. Sci..

[11] Jeyaguru S., Thiagamani S.M.K., Siengchin S., Subramanian J., Ebrahimnezhad-Khaljiri H., Sanjay M.R., Khan A., Abuthakeer S.S., Rajesh S., Alromaizan A.N. (2024). Effect of various weaving architectures on mechanical, vibration and acoustic behavior of Kevlar-Hemp intra-ply hybrid composites. Compos. Part A Appl. Sci. Manuf..

[12] Ozair H., Rehman M.A.U., Baluch A.H., Yaqoob K., Qazi I., Wadood A. (2022). Impact Energy Absorption Analysis of Shape Memory Hybrid Composites. J. Compos. Sci..

[13] Chen X., Zhou Y., Wells G. (2014). Numerical and experimental investigations into ballistic performance of hybrid fabric panels. Compos. Part B Eng..

[14] Iftekhar H., Umair M., Hamdani S.T.A., Imran S.M., Nazir M.S., Ali Z. (2023). Effect of Hybrid Weave Patterns on the Mechanical Performance of Woven Fabrics. J. Nat. Fibers.

[15] Pranesh K.G., Attel M., Nagaraja K.C., Raghavendra B., Prajwal D., Abhijit B., Chander P. (2024). Influence of Nanosilica on Mechanical Performance in Woven Carbon/Kevlar/Epoxy Hybrid Composites. J. Eng..

[16] Naveen Kumar G., Rajesh K., Rama Durga Rao M., Sai Bharath K.P., Eswara Manikanta J. (2023). A review on mechanical properties of hybrid polymer composites. Mater. Today Proc..

[17] Premnath K., Arunprasath K., Sanjeevi R., Elilvanan R., Ramesh M. (2024). Natural/synthetic fiber reinforced hybrid composites on their mechanical behaviors—A review. Interactions.

[18] Aisyah H.A., Paridah M.T., Khalina A., Sapuan S.M., Wahab M.S., Berkalp O.B., Lee C.H., Lee S.H. (2018). Effects of Fabric Counts and Weave Designs on the Properties of Laminated Woven Kenaf/Carbon Fibre Reinforced Epoxy Hybrid Composites. Polymers.

[19] Murat B.I.S., Rahman A.A.A. (2017). Study of Impact Damage Behavior in Woven Carbon Fiber Plates. Procedia Eng..

[20] Aisyah H.A., Paridah M.T., Sapuan S.M., Ilyas R.A., Khalina A., Nurazzi N.M., Lee S.H., Lee C.H. (2021). A Comprehensive Review on Advanced Sustainable Woven Natural Fibre Polymer Composites. Polymers.

[21] Wielhorski Y., Mendoza A., Rubino M., Roux S. (2022). Numerical modeling of 3D woven composite reinforcements: A review. Compos. Part A Appl. Sci. Manuf..

[22] Safdar M.M., Khan M.I., Alsunbul M., Fayad E., Rehan Z.A., Ullah T., Shakir H.M.F., Umair M. (2024). Effect of hybrid weaving patterns on mechanical performance of 3D woven structures. Polym. Polym. Compos..

[23] Chowdhury S., Tripathi L., Behera B.K. (2024). Review: Impact resistance and damage tolerance of 3D woven composites. J. Mater. Sci..

[24] Page M.J., McKenzie J.E., Bossuyt P.M., Boutron I., Hoffmann T.C., Mulrow C.D., Shamseer L., Tetzlaff J.M., Akl E.A., Brennan S.E. (2021). The PRISMA 2020 statement: An updated guideline for reporting systematic reviews. Syst. Rev..

[25] Scopus Database. https://www.scopus.com.

[26] van Eck N.J., Waltman L. (2010). Software survey: VOSviewer, a computer program for bibliometric mapping. Scientometrics.

[27] Ashok Kumar B., Saminathan R., Tharwan M., Vigneshwaran M., Sekhar Babu P., Ram S., Manoj Kumar P. (2022). Study on the mechanical properties of a hybrid polymer composite using egg shell powder based bio-filler. Mater. Today Proc..

[28] Gul S., Tabrizi I.E., Okan B.S., Kefal A., Yildiz M. (2021). An experimental investigation on damage mechanisms of thick hybrid composite structures under flexural loading using multi-instrument measurements. Aerosp. Sci. Technol..

[29] Chen D.D., Sun G.Y., Meng M.Z., Jin X.H., Li Q. (2019). Flexural performance and cost efficiency of carbon/basalt/glass hybrid FRP composite laminates. Thin-Walled Struct..

[30] Rezasefat M., Gonzalez-Jimenez A., Ma D., Vescovini A., Lomazzi L., da Silva A.A.X., Amico S.C., Manes A. (2022). Experimental study on the low-velocity impact response of inter-ply S2-glass/aramid woven fabric hybrid laminates. Thin-Walled Struct..

[31] Chowdhury S., Dubey D., Behera B.K. (2024). An experimental investigation on the influence of weave architectures on out-of-plane and in-plane impact properties of 3D woven fabric-reinforced composites. J. Mater. Sci..

[32] Patel D.K., Waas A.M., Yen C.-F. (2019). Compressive response of hybrid 3D woven textile composites (H3DWTCs): An experimentally validated computational model. J. Mech. Phys. Solids.

[33] Tai J., Wu C., Han G., Sun D., Zhao G., Chen Q., Liu Y. (2024). Study on ballistic penetration behavior of unidirectional UHMWPE/carbon fiber composites: Experiments and numerical modeling. Polym. Compos..

[34] Xu W., Zikry M., Seyam A.-F.M. (2024). Numerical Study of the Influence of the Structural Parameters on the Stress Dissipation of 3D Orthogonal Woven Composites under Low-Velocity Impact. Technologies.

[35] Ferreira L.M., Coelho C., Reis P.N.B. (2023). Numerical Simulations of the Low-Velocity Impact Response of Semicylindrical Woven Composite Shells. Materials.

[36] Taherzadeh-Fard A., Cornejo A., Jiménez S., Barbu L.G. (2025). Numerical Analysis of Damage in Composites: From Intra-Layer to Delamination and Data-Assisted Methods. Mathematics.

[37] Saha S., Das S., Rahman M.Z. (2026). Hybridization in natural fiber composites: Enhanced performance and sustainability. Compos. Part B Eng..

[38] Biswas W., Dong C. (2024). Eco-Efficiency Performance for Multi-Objective Optimal Design of Carbon/Glass/Flax Fibre-Reinforced Hybrid Composites. Sustainability.

[39] Ntsie O.D., Phiri R., Boonyasopon P., Rangappa S.M., Siengchin S. (2025). Advancing sustainable infrastructure: Natural fiber-reinforced composites in engineering. Discov. Appl. Sci..

[40] Daneshjo N., Sabadka D., Malega P. (2024). A Real Test and Simulation Result Comparison of Selected Properties of Hybrid Composite Materials. Eng. Technol. Appl. Sci. Res..

[41] Cheng Z.-Q., Liu H., Tan W. (2024). Advanced computational modelling of composite materials. Eng. Fract. Mech..

[42] Gangwar A., Kumar V., Yaylaci M., Panda S.K. (2024). Computational Modelling and Mechanical Characteristics of Polymeric Hybrid Composite Materials: An Extensive Review. Arch. Comput. Methods Eng..

[43] He C., Ge J., Zhang B., Gao J., Zhong S., Liu W.K., Fang D. (2020). A hierarchical multiscale model for the elastic-plastic damage behavior of 3D braided composites at high temperature. Compos. Sci. Technol..

[44] Liu Z., Wang H., Yang L., Du J. (2022). Research on mechanical properties and durability of flax/glass fiber bio-hybrid FRP composites laminates. Compos. Struct..

[45] Zeeshan M., Ali M., Anjum A.S., Nawab Y. (2021). Optimization of mechanical/thermal properties of glass/flax/waste cotton hybrid composite. J. Ind. Text..

[46] Altaee M.A., Mostafa N.H. (2023). Mechanical properties of interply and intraply hybrid laminates based on jute-glass/epoxy composites. J. Eng. Appl. Sci..

[47] Das S.C., La Rosa A.D., Goutianos S., Grammatikos S. (2024). Glass fibre hybridization to improve the durability of circular flax fibre reinforced composites with off-the-shelf recyclable polymer matrix systems for large scale structural applications. Compos. Part C Open Access.

[48] Kaufmann J., Temesgen A.G., Cebulla H. (2025). A comprehensive review on natural fiber reinforced hybrid composites processing techniques, material properties and emerging applications. Discov. Mater..

[49] Raj M.K.A., Kumar P.M., Palanisamy P., Dharmaraju S., Periyasamy S., Palaniappan M., Gebreyohannes D.T. (2025). Study on the mechanical characteristics of a natural Fiber-based hybrid polymer composite. Sci. Rep..

[50] Mochane M.J., Mokhena T.C., Mokhothu T.H., Mtibe A., Sadiku E.R., Ray S.S., Ibrahim I.D., Daramola O.O. (2019). Recent progress on natural fiber hybrid composites for advanced applications: A review. Express Polym. Lett..

[51] Bunpheng W., Dhairiyasamy R., Varshney D., Singh S., Chan C.K. (2025). Reducing Material Footprint Through Hybrid Bio-Synthetic Polymer Composites: Advanced Testing and Predictive Modeling Approaches. J. Compos. Sci..

[52] Weatherburn A., Montgomery C., Scott G., Ralph C., Girkin J., McGarrigle C., McIlhagger A., Archer E., Szyniszewski S. (2025). Biomimetic polypropylene-carbon intra-ply hybrid 3D woven composite with enhanced impact resistance. Compos. Struct..

[53] (2023). Plastics-Determination of Tensile Properties—Part 4: Test Conditions for Isotropic and Orthotropic Fibre-reinforced Plastic Composites.

[54] (1998). Fibre-reinforced Plastic Composites—Determination of Flexural Properties.

[55] (2023). Fibre-Reinforced Plastic Composites—Determination of Compressive Properties in the In-Plane Direction.

[56] (1997). Fibre-Reinforced Plastic Composites—Determination of the In-Plane Shear Stress/Shear Strain Response, Including the In-Plane Shear Modulus and Strength, by the ±45° Tension Test Method.

[57] (2009). Carbon-Fibre-Reinforced Plastics—Determination of Compression-After-Impact Properties at a Specified Impact-Energy Level.

[58] Rajpurohit A., Joannès S., Singery V., Sanial P., Laiarinandrasana L. (2020). Hybrid Effect in In-Plane Loading of Carbon/Glass Fibre Based Inter- and Intraply Hybrid Composites. J. Compos. Sci..

[59] Arkuszynski P., Roskowicz M. (2025). Influence of Preloading on Damage in CFRP Composite Material Subjected to Low-Energy Impact Loads. Materials.

[60] Sayam A., Rahman A.N.M.M., Rahman M.S., Smriti S.A., Ahmed F., Rabbi M.F., Hossain M., Faruque M.O. (2022). A review on carbon fiber-reinforced hierarchical composites: Mechanical performance, manufacturing process, structural applications and allied challenges. Carbon Lett..

[61] Begum M.S., Milašius R. (2022). Factors of Weave Estimation and the Effect of Weave Structure on Fabric Properties: A Review. Fibers.

[62] Tewani H., Cyvas J., Perez K., Prabhakar P. (2025). Arχi-Textile composites: Role of weave architecture on mode-I fracture energy in woven composites. Compos. Part A Appl. Sci. Manuf..

[63] Sahbaz Karaduman N. (2022). Experimental investigation of the effect of weave type on the mechanical properties of woven hemp fabric/epoxy composites. J. Compos. Mater..

[64] Huang J., Boisse P., Hamila N., Gnaba I., Soulat D., Wang P. (2021). Experimental and numerical analysis of textile composite draping on a square box. Influence of the weave pattern. Compos. Struct..

[65] Axinte A., Ungureanu D., Țăranu N., Bejan L., Isopescu D.N., Lupășteanu R., Hudișteanu I., Roșca V.E. (2022). Influence of Woven-Fabric Type on the Efficiency of Fabric-Reinforced Polymer Composites. Materials.

[66] Lei Z.X., Ma J., Sun W.K., Yin B.B., Liew K.M. (2023). Low-velocity impact and compression-after-impact behaviors of twill woven carbon fiber/glass fiber hybrid composite laminates with flame retardant epoxy resin. Compos. Struct..

[67] Ma Z., Zhang P., Zhu J. (2022). Influence of fabric structure on the tensile and flexural properties of three-dimensional angle-interlock woven composites. J. Ind. Text..

[68] Chen Z., Pan S., Zhou Z., Lei T., Dong B., Xu P. (2019). The Effect of Shear Deformation on Permeability of 2.5D Woven Preform. Materials.

[69] Rehra J., Jungbluth J., Katri B., Schmeer S., Gurka M., Balle F., Breuer U.P. (2024). Damage and failure mechanisms of hybrid carbon fiber and steel fiber reinforced polymer composites. Compos. Part A Appl. Sci. Manuf..

[70] Srinivas M., Srikanth I., RamaRao G., Swami Naidu G. (2019). Effect of Volume Fraction of Reinforcement Layers on the Mechanical Properties of S-glass-Carbon-Epoxy Hybrid Composites. Mater. Today Proc..

[71] Thianwiboon M. (2019). Optimization of a Hybrid Carbon/Glass Composites Afterbody of the Amphibious Plane with Finite Element Analysis. Eng. J..

[72] Burley A., Aitharaju V. (2023). Enhanced ductility in in-layer glass-carbon fiber/epoxy hybrid composites produced via tailored fiber placement. Compos. Part A Appl. Sci. Manuf..

[73] Goyal A., Melenka G.W. (2023). Investigation of carbon-aramid hybrid braided composites using digital volume correlation. Compos. Struct..

[74] Titire L., Muntenita C., Chivu M. (2024). Impact Resistance of Layered Aramid Fabric: A Numerical Study on Projectile-Induced Damage. Polymers.

[75] He A., Xing T., Liang Z., Luo Y., Zhang Y., Wang M., Huang Z., Bai J., Wu L., Shi Z. (2024). Advanced Aramid Fibrous Materials: Fundamentals, Advances, and Beyond. Adv. Fiber Mater..

[76] Jiang L., Wang J., Xiao S., Li Y., Yang L. (2023). Dynamic tensile properties of carbon/glass hybrid fibre composites under intermediate strain rates via DIC and SEM technology. Thin-Walled Struct..

[77] Hannah T., Martin V., Ellis S., Kraft R.H. (2024). High Speed Impact Testing of UHMWPE Composite Using Orthogonal Arrays. Exp. Mech..

[78] Yang Y., Chen X. (2017). Investigation of failure modes and influence on ballistic performance of Ultra-High Molecular Weight Polyethylene (UHMWPE) uni-directional laminate for hybrid design. Compos. Struct..

[79] Zhao Z.-N., Han B., Zhang R., Zhang Q., Zhang Q.-C., Ni C.-Y., Lu T.J. (2021). Enhancement of UHMWPE encapsulation on the ballistic performance of bi-layer mosaic armors. Compos. Part B Eng..

[80] Chowdhury S.C., Sockalingam S., Gillespie J.W. (2020). Inter-molecular interactions in ultrahigh molecular weight polyethylene single crystals. Comput. Mater. Sci..

[81] Hassoon O.H., Abed M.S., Oleiwi J.K., Tarfaoui M. (2022). Experimental and numerical investigation of drop weight impact of aramid and UHMWPE reinforced epoxy. J. Mech. Behav. Mater..

[82] Özsoy M.İ., Fidan S., Bora M.Ö., Ürgün S. (2025). Understanding the Damage Mechanisms of Basalt/Carbon Fiber Hybrid Composites Under Quasi-Static and Dynamic Loadings. Polymers.

[83] Chen D., Luo Q., Meng M., Li Q., Sun G. (2019). Low velocity impact behavior of interlayer hybrid composite laminates with carbon/glass/basalt fibres. Compos. Part B Eng..

[84] Bahrami M., Abenojar J., Martínez M.Á. (2020). Recent Progress in Hybrid Biocomposites: Mechanical Properties, Water Absorption, and Flame Retardancy. Materials.

[85] Attia M.A., El-baky M.A.A., Abdelhaleem M.M., Hassan M.A. (2022). Hybrid composite laminates reinforced with flax-basalt-glass woven fabrics for lightweight load bearing structures. J. Ind. Text..

[86] Khalid M.Y., Arif Z.U., Sheikh M.F., Nasir M.A. (2021). Mechanical characterization of glass and jute fiber-based hybrid composites fabricated through compression molding technique. Int. J. Mater. Form..

[87] Calabrese L., Badagliacco D., Sanfilippo C., Fiore V. (2023). Flax–Glass Fiber Reinforced Hybrid Composites Exposed to a Salt-Fog/Dry Cycle: A Simplified Approach to Predict Their Performance Recovery. Polymers.

[88] Pavlovic A., Valzania L., Minak G. (2025). Effects of Moisture Absorption on the Mechanical and Fatigue Properties of Natural Fiber Composites: A Review. Polymers.

[89] Sahu P., Sharma N., Panda S.K. (2021). Multi-layer advanced fiber hybridisation (glass-carbon-Kevlar) and variable stifiness effect on composite structure responses (stress and deformation): An FE approach. Sci. Iran..

[90] Shah S.Z.H., Megat-Yusoff P.S.M., Karuppanan S., Choudhry R.S., Ahmad F., Sajid Z. (2022). Mechanical Properties and Failure Mechanisms of Novel Resin-infused Thermoplastic and Conventional Thermoset 3D Fabric Composites. Appl. Compos. Mater..

[91] Li Y., Wang F., Shi X., Guo L., Huang C. (2023). Impact Response of 3D Orthogonal Woven Composites with Different Fiber Types. Appl. Compos. Mater..

[92] Fairlie G., Njuguna J. (2020). Damping Properties of Flax/Carbon Hybrid Epoxy/Fibre-Reinforced Composites for Automotive Semi-Structural Applications. Fibers.

[93] Gao H., Li Y. (2025). Dynamic mechanical response evaluation of woven carbon fiber reinforced rubber laminated composites under high strain rates. Sci. Rep..

[94] Poulet T., Bracq A., Demarty Y., Lauro F., Bahlouli N. (2023). Strain rate sensitivity of the in-plane mechanical properties of two UHMWPE thin ply composites. Polym. Test..

[95] Hosur M.V., Adya M., Alexander J., Jeelani S., Vaidya U., Mayer A. (2003). Studies on Impact Damage Resistance of Affordable Stitched Woven Carbon/Epoxy Composite Laminates. J. Reinf. Plast. Compos..

[96] Aziz A.R., Al Abdouli H., Kakur N., Ramos H., Savioli R., Guan Z., Santiago R. (2025). The effects of high strain-rate and temperature on tensile properties of UHMWPE composite laminates. Compos. Part C Open Access.

[97] Weng F., Fang Y., Ren M., Sun J., Feng L. (2021). Effect of high strain rate on shear properties of carbon fiber reinforced composites. Compos. Sci. Technol..

[98] Wu Z., Zhang L., Ying Z., Ke J., Hu X. (2020). Low-velocity impact performance of hybrid 3D carbon/glass woven orthogonal composite: Experiment and simulation. Compos. Part B Eng..

[99] Zhang X., Shi Y., Li Z.-X. (2019). Experimental study on the tensile behavior of unidirectional and plain weave CFRP laminates under different strain rates. Compos. Part B Eng..

[100] Meireman T., Verboven E., Kersemans M., Van Paepegem W., De Clerck K., Daelemans L. (2024). Low-Velocity Impact Resistance and Compression After Impact Strength of Thermoplastic Nanofiber Toughened Carbon/Epoxy Composites with Different Layups. Polymers.

[101] Kravchenko S.G., Volle C., Kravchenko O.G. (2021). An experimental investigation on low-velocity impact response and compression after impact of a stochastic, discontinuous prepreg tape composite. Compos. Part A Appl. Sci. Manuf..

[102] Li Y.M., Jin Y.X., Chang X.T., Shang Y., Cai D.A. (2024). On Low-Velocity Impact Response and Compression after Impact of Hybrid Woven Composite Laminates. Coatings.

[103] Shi X., Sun Y., Xu J., Chen L., Zhang C., Zhang G. (2023). Effect of Fiber Fraction on Ballistic Impact Behavior of 3D Woven Composites. Polymers.

[104] Ding Y., Liu J., Hall Z.E.C., Brooks R.A., Liu H., Kinloch A.J., Dear J.P. (2023). Damage and energy absorption behaviour of composite laminates under impact loading using different impactor geometries. Compos. Struct..

[105] Wang Z., Xian G. (2023). Impact performances of fiber reinforced polymer composites and cables: A review. Compos. Struct..

[106] Zhang Z., Guo H., Lan Y., Zhao L. (2025). Impact Resistance Study of Fiber–Metal Hybrid Composite Laminate Structures: Experiment and Simulation. Materials.

[107] Bunea M., Vizureanu P. (2024). Damage Mechanisms and Mechanical Behavior of Epoxy Composites after Low Velocity Impact. Composite Materials-Science and Engineering.

[108] Shah S.Z.H., Megat-Yusoff P.S.M., Karuppanan S., Choudhry R.S., Ahmad F., Sajid Z., Gerard P., Sharp K. (2021). Performance comparison of resin-infused thermoplastic and thermoset 3D fabric composites under impact loading. Int. J. Mech. Sci..

[109] Sonnenfeld C., Mendil-Jakani H., Agogué R., Nunez P., Beauchêne P. (2017). Thermoplastic/thermoset multilayer composites: A way to improve the impact damage tolerance of thermosetting resin matrix composites. Compos. Struct..

[110] Shah S.Z.H., Megat-Yusoff P.S.M., Karuppanan S., Choudhry R.S., Ud Din I., Othman A.R., Sharp K., Gerard P. (2021). Compression and buckling after impact response of resin-infused thermoplastic and thermoset 3D woven composites. Compos. Part B Eng..

[111] Zhao Y., Cao M., Lum W.P., Tan V.B.C., Tay T.E. (2018). Interlaminar fracture toughness of hybrid woven carbon-Dyneema composites. Compos. Part A Appl. Sci. Manuf..

[112] Broughton W.R., Sims G.D., Forde M. (2009). Chapter 53: Testing and evaluation of polymer composites. ICE Manual of Construction Materials: Volume I: Fundamentals and Theory; Concrete; Asphalts in Road Construction; Masonry.

[113] Hung P.-y., Lau K.-t., Cheng L.-k., Leng J., Hui D. (2018). Impact response of hybrid carbon/glass fibre reinforced polymer composites designed for engineering applications. Compos. Part B Eng..

[114] Ahmed R., Manik K.H., Nath A., Shohag J.R., Mim J.J., Hossain N. (2025). Recent advances in sustainable natural fiber composites: Environmental benefits, applications, and future prospects. Mater. Today Sustain..

[115] Katunin A., Danek W., Wronkowicz A., Dragan K. (2023). Methodology of residual strength prediction of composite structures with low-velocity impact damage based on NDT inspections and numerical-experimental CAI testing. Int. J. Impact Eng..

[116] Katunin A., Wronkowicz-Katunin A., Danek W., Wyleżoł M. (2021). Modeling of a realistic barely visible impact damage in composite structures based on NDT techniques and numerical simulations. Compos. Struct..

[117] Arkuszyński P., Rośkowicz M., Angelika A. (2026). The Application of a Non-Newtonian Fluid as a Protective Layer for a CFRP Material Subjected to Low-Energy Impact Loads. Materials.

[118] Wronkowicz-Katunin A., Katunin A., Dragan K. (2019). Reconstruction of Barely Visible Impact Damage in Composite Structures Based on Non-Destructive Evaluation Results. Sensors.

[119] El-Dessouky H.M., Saleh M.N., Wang Y., Alotaibi M.S. (2021). Effect of Unit-Cell Size on the Barely Visible Impact Damage in Woven Composites. Appl. Sci..

[120] Janeliukstis R., Baranovskis D., Katunin A., Zorin I., Burgholzer P., Lopes H., Dragan K., Rucevskis S., Gaile L., Chen X. (2025). Nondestructive evaluation of barely visible impact damage in composite structures—A review. Compos. Struct..

[121] Torbali M.E., Zolotas A., Avdelidis N.P., Ibarra-Castanedo C., Maldague X.P. (2026). Multi-modal analysis of barely visible impact damage in carbon fibre composites through the fusion of Pulsed Thermography and Phased Array Ultrasonic Testing. Quant. InfraRed Thermogr. J..

[122] Ni K., Chen Q., Wen J., Cai Y., Zhu Z., Li X. (2024). Low-velocity impact and post-impact compression properties of carbon/glass hybrid yacht composite materials. Ocean Eng..

[123] Liu J., Wei X. (2021). Enhancing the impact performance of reinforced composites through fiber hybridization—A hybrid dynamic shear-lag model. Extrem. Mech. Lett..

[124] Raheem A., Subbaya K.M. (2023). Performance evaluation of hybrid polymer composite materials in marine applications: A review. Mater. Today Proc..

[125] Sanchez-Saez S., Barbero E., Zaera R., Navarro C. (2005). Compression after impact of thin composite laminates. Compos. Sci. Technol..

[126] Mathivanan N.R., Jerald J. (2015). Experimental Investigation of Woven E-Glass Epoxy Composite Laminates Subjected to Low-Velocity Impact at Different Energy Levels. J. Miner. Mater. Charact. Eng..

[127] Glud J.A., Carraro P.A., Quaresimin M., Dulieu-Barton J.M., Thomsen O.T., Overgaard L.C.T. (2018). A damage-based model for mixed-mode crack propagation in composite laminates. Compos. Part A Appl. Sci. Manuf..

[128] Zaini M., Hamlaoui O., Chafiq J., El Fqih M.A., Idiri M., Aqil S., Hajji M.K., Bal A., Tozan H., Harnicárová M. (2025). Post-Curing Effects on the Tensile Properties of Hybrid Fiber-Reinforced Polymers: Experimental and Numerical Insights. Polymers.

[129] Lamon F., Maragoni L., Carraro P.A., Quaresimin M. (2023). Fatigue damage evolution in woven composites with different architectures. Int. J. Fatigue.

[130] Oliveira T.L.L., Hadded M., Mimouni S., Schaan R.B. (2025). The Role of Non-Destructive Testing of Composite Materials for Aerospace Applications. NDT.

[131] Tsivolas E., Gergidis L.N., Paipetis A.S. (2022). Crack Growth and Delamination Analysis in GFRP Composite Materials. Appl. Sci..

[132] Yun K., Kwak S., Wang Z., Chang M., Kim J., Liu J., Ri C. (2019). A Damage Model Reflecting the Interaction between Delamination and Intralaminar Crack for Failure Analysis of FRP Laminates. Appl. Sci..

[133] Saidane E.H., Scida D., Pac M.-J., Ayad R. (2019). Mode-I interlaminar fracture toughness of flax, glass and hybrid flax-glass fibre woven composites: Failure mechanism evaluation using acoustic emission analysis. Polym. Test..

[134] Vieille B., Gonzalez J.-D., Bouvet C. (2018). Fracture mechanics of hybrid composites with ductile matrix and brittle fibers: Influence of temperature and constraint effect. J. Compos. Mater..

[135] Tai J.L., Sultan M.T.H., Łukaszewicz A., Józwik J., Oksiuta Z., Shahar F.S. (2025). Recent Trends in Non-Destructive Testing Approaches for Composite Materials: A Review of Successful Implementations. Materials.

[136] Katunin A., Wronkowicz-Katunin A., Dragan K. (2020). Impact Damage Evaluation in Composite Structures Based on Fusion of Results of Ultrasonic Testing and X-ray Computed Tomography. Sensors.

[137] Amif M.A., Jack D.A. (2024). High-Resolution Ultrasound to Quantify Sub-Surface Wrinkles in a Woven CFRP Laminate. Materials.

[138] Farmaki S., Exarchos D.A., Tragazikis I.K., Matikas T.E., Dassios K.G. (2020). A Novel Infrared Thermography Sensing Approach for Rapid, Quantitative Assessment of Damage in Aircraft Composites. Sensors.

[139] Ma M., Wang Z., Gao Z., Jiang M. (2025). Ultrasonic Phased Array Testing and Identification of Multiple-Type Internal Defects in Carbon Fiber Reinforced Plastics Based on Convolutional Neural Network. Materials.

[140] Alhammad M., Avdelidis N.P., Ibarra-Castanedo C., Torbali M.E., Genest M., Zhang H., Zolotas A., Maldgue X.P.V. (2022). Automated Impact Damage Detection Technique for Composites Based on Thermographic Image Processing and Machine Learning Classification. Sensors.

[141] Hidayat Z., Avdelidis N.P., Fernandes H. (2025). Brief Review of Vibrothermography and Optical Thermography for Defect Quantification in CFRP Material. Sensors.

[142] Duchene P., Chaki S., Ayadi A., Krawczak P. (2018). A review of non-destructive techniques used for mechanical damage assessment in polymer composites. J. Mater. Sci..

[143] Rashidi A., Olfatbakhsh T., Crawford B., Milani A.S. (2020). A Review of Current Challenges and Case Study toward Optimizing Micro-Computed X-Ray Tomography of Carbon Fabric Composites. Materials.

[144] Chen J., Yu Z., Jin H. (2022). Nondestructive testing and evaluation techniques of defects in fiber-reinforced polymer composites: A review. Front. Mater..

[145] Evans E.E., Brooks R.A., Liu J., Hall Z.E.C., Liu H., Lowe T.J.E., Withers P.J., Kinloch A.J., Dear J.P. (2024). Comparison of X-ray Computed Tomography and Ultrasonic C-Scan Techniques and Numerical Modelling of Impact Damage in a CFRP Composite Laminate. Appl. Compos. Mater..

[146] Xu W., Zikry M., Seyam A.-F.M. (2024). Impact Performance of 3D Orthogonal Woven Composites: A Finite Element Study on Structural Parameters. J. Compos. Sci..

[147] Mutsuddy S., Cai D.a., Hossain M.H., Wang X. (2025). Repeated Low-Velocity Impact Properties of Hybrid Woven Composite Laminates. Materials.

[148] Han L., Qi H., Yang J., Chu F., Lin C., Liu P., Zhang Q. (2024). Study on the Low-Velocity Impact Response and Damage Mechanisms of Thermoplastic Composites. Polymers.

[149] Zhang J., Zhang Z., Huang R., Tan L. (2025). Advances in Toughening Modification Methods for Epoxy Resins: A Comprehensive Review. Polymers.

[150] Mousavi S.R., Estaji S., Raouf Javidi M., Paydayesh A., Khonakdar H.A., Arjmand M., Rostami E., Jafari S.H. (2021). Toughening of epoxy resin systems using core–shell rubber particles: A literature review. J. Mater. Sci..

[151] Domun N., Hadavinia H., Zhang T., Sainsbury T., Liaghat G.H., Vahid S. (2015). Improving the fracture toughness and the strength of epoxy using nanomaterials—A review of the current status. Nanoscale.

[152] Duan Z., Li W., Liu H., Shen P., Yang H., Zhong X., Bao J. (2025). Synergetic Improvement of Interfacial Performance and Impact Resistance of Carbon Fiber-Reinforced Epoxy Composite via Continuous Electrochemical Oxidation. Polymers.

[153] Yang T., Zhao Y., Liu H., Sun M., Xiong S. (2021). Effect of Sizing Agents on Surface Properties of Carbon Fibers and Interfacial Adhesion of Carbon Fiber/Bismaleimide Composites. ACS Omega.

[154] Garg R., Babaei I., Paolino D.S., Vigna L., Cascone L., Calzolari A., Galizia G., Belingardi G. (2020). Predicting Composite Component Behavior Using Element Level Crashworthiness Tests, Finite Element Analysis and Automated Parametric Identification. Materials.

[155] Qiang X., Wang T., Xue H., Ding J., Deng C. (2024). Study on Low-Velocity Impact and Residual Compressive Mechanical Properties of Carbon Fiber–Epoxy Resin Composites. Materials.

[156] Yu J., Huang J., Zhang L., Guo L. (2025). Multiscale analysis of the hybridization influence on the failure mechanisms of 2D woven composites. Eng. Fract. Mech..

[157] Akshat T., Petru M., Mishra R.K. (2025). Low Velocity Drop-Weight Impact of Flax–Glass Hybrid Composites for Application in Automotive Components: Numerical Modelling and Experimental Analysis. Materials.

[158] Damghani M., Ersoy N., Piorkowski M., Murphy A. (2019). Experimental evaluation of residual tensile strength of hybrid composite aerospace materials after low velocity impact. Compos. Part B Eng..

[159] Farokhi Nejad A., Bin Salim M.Y., Rahimian Koloor S.S., Petrik S., Yahya M.Y., Abu Hassan S., Mohd Shah M.K. (2021). Hybrid and Synthetic FRP Composites under Different Strain Rates: A Review. Polymers.

[160] Ferreira L.M., Coelho C.A.C.P., Reis P.N.B. (2023). Numerical predictions of intralaminar and interlaminar damage in thin composite shells subjected to impact loads. Thin-Walled Struct..

[161] Liu C., Xie J., Sun Y., Chen L. (2019). Micro-scale modeling of textile composites based on the virtual fiber embedded models. Compos. Struct..

[162] Yang N., Zou Z., Soutis C., Potluri P., Katnam K.B. (2025). Effect of Yarn-Level Fibre Hybridisation on Thermomechanical Behaviour of 3D Woven Orthogonal Flax/E-Glass Composite Laminae. J. Compos. Sci..

[163] Falaschetti M.P., Rondina F., Zavatta N., Troiani E., Donati L. (2024). Effective implementation of numerical models for the crashworthiness of composite laminates. Eng. Fail. Anal..

[164] de Oliveira Filho G.C., de Sousa Mota R.C., da Conceicao A.C.R., Leao M.A., de Araujo Filho O.O. (2019). Effects of hybridization on the mechanical properties of composites reinforced by piassava fibers tissue. Compos. Part B Eng..

[165] Raimondi L., Salvi L., Semprucci F., Falaschetti M.P. (2025). Modelling and Validation of Progressive Damage in Hybrid CFRP–Elastomer Laminates Under Quasi-Static Indentation Loading. Appl. Sci..

[166] Millen S.L.J., Lee J. (2023). Microscale modelling of lightning damage in fibre-reinforced composites. J. Compos. Mater..

[167] Balasubramani N.K., Zhang B., Chowdhury N.T., Mukkavilli A., Suter M., Pearce G.M. (2022). Micro-mechanical analysis on random RVE size and shape in multiscale finite element modelling of unidirectional FRP composites. Compos. Struct..

[168] Ye J., Cai H., Liu L., Zhai Z., Amaechi C.V., Wang Y., Wan L., Yang D., Chen X., Ye J. (2021). Microscale intrinsic properties of hybrid unidirectional/woven composite laminates: Part I experimental tests. Compos. Struct..

[169] Li B., Liu C., Zhao X., Ye J., Guo F. (2023). Multiscale Study of the Effect of Fiber Twist Angle and Interface on the Viscoelasticity of 2D Woven Composites. Materials.

[170] Ghosh G., Biswas D., Bhattacharyya R. (2025). Advancements in multiscale modeling of damage in composite materials: A comprehensive review. Compos. Part B Eng..

[171] Júnior C.J.F., Nandurdikar V., Neto A.G., Harish A.B. (2024). Concurrent multiscale modelling of woven fabrics: Using beam finite elements with contact at mesoscale. Finite Elem. Anal. Des..

[172] Shi J., Zhang J., Du K., Guo Q., Hou Y., Dong C. (2024). A Multiscale Modeling and Experimental Study on the Tensile Strength of Plain-Woven Composites with Hybrid Bonded–Bolted Joints. Polymers.

[173] Heide-Jørgensen S., Budzik M.K., Ibsen C.H. (2022). Three-dimensional, multiscale homogenization for hybrid woven composites with fiber-matrix debonding. Compos. Sci. Technol..

[174] Li K., Qian Y., Cheng S., Yang Z., Lu Z. (2025). Multi-scale damage modeling and failure mechanisms analysis of 3D woven composites with considering stochastic fiber strength and initial misalignment angle dispersion. Compos. Part A Appl. Sci. Manuf..

[175] Pham D.C., Cui X., Ren X., Lua J. (2019). A discrete crack informed 3D continuum damage model and its application for delamination migration in composite laminates. Compos. Part B Eng..

[176] Liu H., Falzon B.G., Tan W. (2018). Experimental and numerical studies on the impact response of damage-tolerant hybrid unidirectional/woven carbon-fibre reinforced composite laminates. Compos. Part B Eng..

[177] Martínez-Hergueta F., Ares D., Ridruejo A., Wiegand J., Petrinic N. (2019). Modelling the in-plane strain rate dependent behaviour of woven composites with special emphasis on the non-linear shear response. Compos. Struct..

[178] Feld N., Coussa F., Delattre B. (2018). A novel approach for the strain rate dependent modelling of woven composites. Compos. Struct..

[179] AllahTavakoli Y., Ichchou M.N., Marquis-Favre C., Hamzaoui N. (2023). On a hybrid updating method for modeling vibroacoustic behaviors of composite panels. J. Sound Vib..

[180] Corrado G., Arteiro A., Marques A.T., Daoud F., Glock F. (2024). Mesoscale Model for Composite Laminates: Verification and Validation on Scaled Un-Notched Laminates. Polymers.

[181] Obert E., Daghia F., Ladevèze P., Ballere L. (2014). Micro and meso modeling of woven composites: Transverse cracking kinetics and homogenization. Compos. Struct..

[182] Sabuncuoglu B., Lomov S.V. (2020). Micro-scale numerical study of fiber/matrix debonding in steel fiber composites. J. Eng. Fibers Fabr..

[183] Ferreira L.M., Coelho C.A.C.P., Reis P.N.B. (2023). Effect of Cohesive Properties on Low-Velocity Impact Simulations of Woven Composite Shells. Appl. Sci..

[184] Zhang Y., Van Paepegem W., De Corte W. (2024). An Enhanced Progressive Damage Model for Laminated Fiber-Reinforced Composites Using the 3D Hashin Failure Criterion: A Multi-Level Analysis and Validation. Materials.

[185] Mazumder A., Liu Q., Wang Y., Yen C.-F. (2022). A non-linear material model for progressive damage analysis of woven composites using a conformal meshing framework. J. Compos. Mater..

[186] Fisher T., Almeida J.H.S., Burhan M., Kazancı Z. (2024). Development of a new progressive damage model for woven fabric composites. Mech. Adv. Mater. Struct..

[187] Ahmed S., Zheng X., Zhang D., Yan L. (2020). Impact Response of Carbon/Kevlar Hybrid 3D Woven Composite Under High Velocity Impact: Experimental and Numerical Study. Appl. Compos. Mater..

[188] Hu X., Tang J., Xiao W., Qu K. (2021). A Strain Rate Dependent Progressive Damage Model for Carbon Fiber Woven Composites under Low Velocity Impact. J. Phys. Conf. Ser..

[189] Sharma H., Singh A. (2025). Combined phase-field and cohesive zone modeling for mixed-mode fracture in polymer composites. Eng. Comput..

[190] Giannopoulos I.K., Yasaee M., Maropakis N. (2021). Ballistic Impact and Virtual Testing of Woven FRP Laminates. J. Compos. Sci..

[191] Baharvand A., Teuwen J.J.E., Shankar Verma A. (2025). A Review of Damage Tolerance and Mechanical Behavior of Interlayer Hybrid Fiber Composites for Wind Turbine Blades. Materials.

[192] Zhu G., Zhang Y., Niu X., Duan C., Wang Z., Zhao X. (2025). Novel multiscale modeling strategy for hybrid fiber reinforced composites. Int. J. Mech. Sci..

[193] Li C., He W., Nie X., Wei X., Guo H., Wu X., Xu H., Zhang T., Liu X. (2021). Intelligent damage recognition of composite materials based on deep learning and ultrasonic testing. AIP Adv..

[194] Wei Z., Fernandes H., Herrmann H.-G., Tarpani J.R., Osman A. (2021). A Deep Learning Method for the Impact Damage Segmentation of Curve-Shaped CFRP Specimens Inspected by Infrared Thermography. Sensors.

[195] Pineda E.J., Bednarcyk B.A., Ricks T.M., Farrokh B., Jackson W. (2022). Multiscale failure analysis of a 3D woven composite containing manufacturing induced voids and disbonds. Compos. Part A Appl. Sci. Manuf..

[196] Ricks T.M., Pineda E.J., Bednarcyk B.A., McCorkle L.S., Miller S.G., Murthy P.L.N., Segal K.N. (2022). Multiscale Progressive Failure Analysis of 3D Woven Composites. Polymers.

[197] Raju K., Tay T.-E., Tan V.B.C. (2021). A review of the FE2 method for composites. Multiscale Multidiscip. Model. Exp. Des..

[198] Ullah Z., Zhou X.Y., Kaczmarczyk L., Archer E., McIlhagger A., Harkin-Jones E. (2019). A unified framework for the multi-scale computational homogenisation of 3D-textile composites. Compos. Part B Eng..

[199] Rehman A., Lathkar G.S., Suryawanshi V.B. (2025). Evaluation of the Mechanical Properties of Hybrid Composites Encompassing Natural Fibers (Flax, Hemp, Kenaf, Jute and Sisal) and Glass Fiber, through Experimental and Numerical Approaches. Int. J. Veh. Struct. Syst..

[200] Ratković J., Ivančević D. (2025). Computationally efficient modelling of impact and perforation in woven FRP composites. Compos. Struct..

[201] Ghane E., Fagerström M., Mirkhalaf S.M. (2023). A multiscale deep learning model for elastic properties of woven composites. Int. J. Solids Struct..

[202] Liu X., Zhou X.-Y., Liu B., Gao C. (2023). Multiscale modeling of woven composites by deep learning neural networks and its application in design optimization. Compos. Struct..

[203] Liu X., Tian S., Tao F., Yu W. (2021). A review of artificial neural networks in the constitutive modeling of composite materials. Compos. Part B Eng..

[204] Fu X., Zhou F., Peddireddy D., Kang Z., Jun M.B.-G., Aggarwal V. (2023). An finite element analysis surrogate model with boundary oriented graph embedding approach for rapid design. J. Comput. Des. Eng..

[205] Zhu K., Cao W., Ran C., Gu B. (2023). A novel automatic crack classification algorithm of 3-D woven composites based on deep-learning U-Net model. Eng. Fract. Mech..

[206] Azad M.M., Shah A.u.R., Prabhakar M.N., Kim H.S. (2024). Deep Learning-Based Microscopic Damage Assessment of Fiber-Reinforced Polymer Composites. Materials.

[207] Reiner J., Linden N., Vaziri R., Zobeiry N., Kramer B. (2023). Bayesian parameter estimation for the inclusion of uncertainty in progressive damage simulation of composites. Compos. Struct..

[208] Hong X., Wang P., Yang W., Zhang J., Chen Y., Li Y. (2024). A multiscale Bayesian method to quantify uncertainties in constitutive and microstructural parameters of 3D-printed composites. J. Mech. Phys. Solids.

[209] Shah S.Z.H., Lee J., Megat-Yusoff P.S.M., Hussain S.Z., Sharif T., Choudhry R.S. (2023). Multiscale damage modelling of notched and un-notched 3D woven composites with randomly distributed manufacturing defects. Compos. Struct..

[210] Wang Z., Hawi P., Masri S., Aitharaju V., Ghanem R. (2023). Stochastic multiscale modeling for quantifying statistical and model errors with application to composite materials. Reliab. Eng. Syst. Saf..

[211] Ye D., Veen L., Nikishova A., Lakhlili J., Edeling W., Luk O.O., Krzhizhanovskaya V.V., Hoekstra A.G. (2021). Uncertainty quantification patterns for multiscale models. Philos. Trans. R. Soc. A Math. Phys. Eng. Sci..

[212] Research E.M. Hybrid Composites Market Report 2026–2035. https://www.marketresearchfuture.com/reports/hybrid-composites-market-6920.

[213] Monteiro B., Simões S. (2024). Recent Advances in Hybrid Nanocomposites for Aerospace Applications. Metals.

[214] Rajak D.K., Pagar D.D., Behera A., Menezes P.L., Kumar V., Agarwal A.K., Jena A., Upadhyay R.K. (2022). Role of Composite Materials in Automotive Sector: Potential Applications. Advances in Engine Tribology.

[215] Wazeer A., Das A., Abeykoon C., Sinha A., Karmakar A. (2023). Composites for electric vehicles and automotive sector: A review. Green Energy Intell. Transp..

[216] Swolfs Y. (2017). Perspective for Fibre-Hybrid Composites in Wind Energy Applications. Materials.

[217] Dong S., Li C., Xian G. (2021). Environmental Impacts of Glass- and Carbon-Fiber-Reinforced Polymer Bar-Reinforced Seawater and Sea Sand Concrete Beams Used in Marine Environments: An LCA Case Study. Polymers.

[218] Rubino F., Nisticò A., Tucci F., Carlone P. (2020). Marine Application of Fiber Reinforced Composites: A Review. J. Mar. Sci. Eng..

[219] Fajri A., Suryanto S., Adiputra R., Prabowo A.R., Tjahjana D.D.D.P., Yaningsih I., Laksono F.B., Nurrohmad A., Nugroho A., Wandono F.A. (2024). Tensile assessment of woven CFRP using finite element method: A benchmarking and preliminary study for thin-walled structure application. Curved Layer. Struct..

[220] Ortega Z., Matarazzo A., Suárez L., Catalfo P., Ingrao C. (2025). Why hybrid composite materials? Findings from a systematic literature review of life cycle assessments. Sustain. Mater. Technol..

[221] Ribeiro F., Correia L., Sena-Cruz J. (2024). Hybridization in FRP Composites for Construction: State-of-the-Art Review and Trends. J. Compos. Constr..

[222] Nguyen H., Zatar W., Mutsuyoshi H., Thakur V.K., Thakur M.K., Pappu A. (2017). Hybrid polymer composites for structural applications. Hybrid Polymer Composite Materials.

[223] Przybek A. (2025). The Role of Natural Fibers in the Building Industry—The Perspective of Sustainable Development. Materials.

[224] Karahan M., Ahrari M., Karahan N. (2023). Composite Materials Market Research and Export Potential Analysis: A Regio-Global Case Study. RECENT-Rezult. Cercet. Noastre Teh..

[225] Shiferaw M., Tegegne A., Asmare A., Mulatie T., Tesfaye S. (2023). An overview of the role of composites in the application of lightweight body parts and their environmental impact: Review. Eng. Solid Mech..

[226] Kumar V. (2024). Hybrid Composite Materials and Manufacturing: Fibers, Nano-Fillers and Integrated Additive Processes.

[227] Zanjani J.S.M., Baran I. (2021). Co-Bonded Hybrid Thermoplastic-Thermoset Composite Interphase: Process-Microstructure-Property Correlation. Materials.

[228] Council N.R. (2012). Application of Lightweighting Technology to Military Aircraft, Vessels, and Vehicles.

[229] Rybicka J., Tiwari A., Leeke G.A. (2016). Technology readiness level assessment of composites recycling technologies. J. Clean. Prod..

[230] Larsen L., Endrass M., Jarka S., Bauer S., Janek M. (2025). Exploring ultrasonic and resistance welding for thermoplastic composite structures: Process development and application potential. Compos. Part B Eng..

[231] Zhao G., Xu T., Fu X., Zhao W., Wang L., Lin J., Hu Y., Du L. (2024). Machine-learning-assisted multiscale modeling strategy for predicting mechanical properties of carbon fiber reinforced polymers. Compos. Sci. Technol..

[232] Arunprasath K., Senthamaraikannan P., Suyambulingam I., Akash S., Kathic S., Chanth M.V., Sunesh N., Kumar R. (2025). From Degradation to Durability: Strategies for Prolonging the Shelf Life of Natural Fiber Composites—A Comprehensive Review. J. Nat. Fibers.

[233] Alblalaihid K.S., Aldoihi S.A., Alharbi A.A. (2024). Structural Health Monitoring of Fiber Reinforced Composites Using Integrated a Linear Capacitance Based Sensor. Polymers.

[234] del Bosque A., Vergara D., Fernández-Arias P. (2025). An Overview of Smart Composites for the Aerospace Sector. Appl. Sci..

[235] Yang L., Wang J., Wang Q., Xiao P., Gao J., Wu Z. (2025). Preparation and performance characterization of intelligent woven composites embedded with distributed fiber optic sensors. Compos. Commun..

